# Integrated epigenomic and transcriptional profiling reveals genotype-specific adaptive reprogramming to drought stress in *Brassica napus*

**DOI:** 10.1186/s12870-026-08405-0

**Published:** 2026-03-02

**Authors:** Wei Huang, Weizhuo Zhu, Zi Zhang, Weiping Ma, Hongyu Zhou, Zhanan Zhou, Shengguan Cai, Dezhi Wu, Lixi Jiang, Tao Yan

**Affiliations:** 1 Institute of Crop Science, Zhejiang University, Yu-Hang-Tang Road 866, Hangzhou, 310058 China; 2 College of Agronomy, Hunan Agriculture University, Changsha, 410128 China

**Keywords:** *Brassica napus*, Drought stress, DNA methylation, Transcriptome, Multi-omics integration, CHH methylation, Stress adaptation

## Abstract

**Background:**

Rapeseed (*Brassica napus* L.) is a globally important oil crop whose productivity is increasingly threatened by drought stress. Although DNA methylation is recognized as a key regulator of plant stress responses, the integrated epigenetic and transcriptional landscapes underlying divergent drought tolerance in rapeseed remain poorly understood.

**Results:**

Here we performed whole-genome bisulfite sequencing (WGBS) and RNA sequencing (RNA-seq) on extreme drought-tolerant and drought-sensitive rapeseed genotypes. Drought stress triggered extensive DNA methylation reprogramming, with differentially methylated regions (DMRs) in the CHH context accounting for over 68% of all significant DMRs, highlighting the prominent role of non-CG methylation. The drought-sensitive genotypes exhibited an “over-defense” strategy, characterized by pervasive hyper-methylation, a larger number of DMRs and differentially expressed genes (DEGs), and widespread activation of stress-response pathways. In contrast, the tolerant genotypes displayed a “precision-regulation” strategy, featuring balanced methylation dynamics, fewer but highly specific DMRs and DEGs, and enrichment of pathways associated with carbohydrate transport and resource allocation. Integrated multi-omics analysis identified 19 core pathways consistently altered at both epigenetic and transcriptional levels. Furthermore, we identified 106 high-confidence candidate genes exhibiting negative correlations between DNA methylation and gene expression, among which 12 hub genes were located within the core pathways, including pivotal regulators such as *BnBBX21* and *BnTAT7*.

**Conclusions:**

Our results reveal two contrasting molecular strategies underlying drought adaptation in rapeseed: an extensive but energetically costly “over-defense” response in sensitive genotypes and a more efficient “precision-regulation” strategy in tolerant genotypes. These strategies are mediated by genotype-specific coordination between epigenetic remodeling and transcriptional reprogramming. This study provides mechanistic insights into drought adaptation and highlights valuable epigenetic and genetics targets for improving drought resilience in rapeseed.

**Supplementary Information:**

The online version contains supplementary material available at 10.1186/s12870-026-08405-0.

## Background

 Rapeseed (*Brassica napus* L.) is a one of the most important oil crops worldwide, contributing approximately 15% of the global supply of edible vegetable oil supply and thereby playing a vital role in ensuring food security [1]. However, rapeseed production is highly vulnerable to drought stress. Under the context of global climate change, the increasing frequency and intensity of drought events have emerged as major constraints on crop productivity. Severe drought stress has been reported to cause yield reductions of 30–50% in major crops [2]. In China, one of the world’s leading rapeseed producers, seasonal autumn drought frequently disrupts seedling establishment and represents a primary factor contributing to yield instability and production losses [3].

In response to drought stress, rapeseed activates a complex network of physiological and molecular mechanisms. At the physiological level, drought inhibits photosynthesis efficiency, induces excessive accumulation of reactive oxygen species (ROS), and ultimately leads to oxidative damage to cellular components [4, 5]. To mitigate these effects, plants deploy a series of adaptive responses, including the accumulation of osmoregulatory substances such as proline, activation of antioxidant enzyme systems (e.g., superoxide dismutase (SOD), and peroxidase (POD)), and extensive reprogramming of phytohormone signaling pathways, particularly those involving abscisic acid (ABA) and jasmonic acid (JA) [6, 7]. These coordinated responses are essential for maintaining cellular homeostasis and survival under drought stress.

Beyond transcriptional regulation, accumulating evidence highlights epigenetic mechanisms, particularly DNA methylation, as central regulators of plant stress responses and stress memory [8, 9]. DNA methylation involves the addition of methyl groups to cytosine bases in different sequence contexts, namely symmetric CG and CHG, and asymmetric CHH (where H denotes A, T, or C), facilitating rapid and reversible regulation of gene expression and transposable element activity without altering the underlying DNA sequence [10]. In *B. napus*, DNA methylation has been implicated in coordinating responses to various abiotic stresses, with non-CG methylation, especially in the CHH context, showing pronounced plasticity under drought conditions [11, 12]. Such dynamic CHH methylation is thought to be particularly important for rapid environmental adaptation [13].

As an allopolyploid species with A and C subgenomes, *B. napus* exhibits pronounced subgenomic asymmetry in gene expression and stress responsiveness [14]. Increasing evidence suggests that the C subgenome plays a dominant role in the epigenetic regulation and environmental adaption [15–17]. Previous studies have reported preferential epigenetic remodeling and stress-responsive gene activation within the C subgenome, providing new insights into the molecular basis of drought tolerance in rapeseed [18, 19]. Moreover, structural variation and regulatory divergence within the C subgenome have been shown to substantially influence gene expression and contribute to environmental adaptation in *B. napus*, underscoring its importance in stress-related phenotypic differentiation [20].

Most existing studies have focus on a single regulatory layer, limiting our understanding of how epigenetic modifications are translated into transcriptional and phenotypic outcomes. Integrating methylome and transcriptome data can more systematically unravel the complete regulatory cascade from epigenetic modification to gene expression, which is crucial for understanding the intrinsic molecular differentiation among extreme drought-tolerant genotypes [21, 22]. Such multi-omics integration has been successfully applied to dissect complex traits in rapeseed, providing a powerful strategy for identifying key genetic regulators [23].

In this study, leveraging prior genome-wide association study (GWAS) results for seedling drought tolerance in *B. napus* [24], we selected extreme drought-tolerant and drought-sensitive genetic accessions for whole-genome bisulfite sequencing (WGBS) and RNA sequencing (RNA-seq). By integrating epigenomic and transcriptomic datasets, we aimed to systematically characterize the regulatory landscape underlying divergent drought responses. Specifically, this study sought to: (i) elucidate the involvement of CHH methylation to drought-induced epigenomic reprogramming; (ii) uncover the molecular basis distinguishing broad “over-defense” responses from more targeted “precision-regulation” strategies; and (iii) identify key hub genes linking DNA methylation dynamics to transcriptional reprogramming. Together, these analyses provide new insights into the multi-layered regulatory network of drought adaptation and offer valuable targets for improving drought resilience in rapeseed.

## Results

### Global DNA methylation landscape and pre-stress epigenomic architecture

To investigate the epigenomic dynamics under drought stress, we performed WGBS on 24 samples from 12 extreme phenotypes (6 tolerant and 6 sensitive) under well-watered (control) and drought conditions. Data quality control confirmed high-quality sequencing suitable for downstream analysis. Each sample yielded an average of 30.94 Gb of high-quality clean data, with a clean data ratio of 88.12% (Table S1). The Q20 (96.54%), Q30 (89.82%) values, average bisulfite conversion rate (99.90%), alignment rate (54.7%) and other quality control metrics all met standards for downstream analysis (Table S2-S3) [25–27].

Analysis of the global DNA methylation pattern in *B. napus* revealed that under control conditions, the tolerant and sensitive materials exhibited highly similar methylation profiles (Fig. S1). As summarized in Table 1, the CG context constituted the largest proportion in both (Sensitive: 56.68%, Tolerant: 57.36%), with average methylation levels exceeding 91%. The proportions and levels of CHG and CHH contexts were also closely comparable. This baseline similarity suggests that differential drought tolerance likely stems from divergent capacities for dynamic epigenomic reprogramming under stress, rather than inherent global methylation states.

**Table 1:** Global methylation features of different *B. napus* phenotypes under control conditions

context	phenotype	methylation_level	percentage_of_total_mC
CG	tolerant	92.30806	56.35855
CHG	tolerant	46.75625	21.14523
CHH	tolerant	32.16392	22.49623
CG	sensitive	91.90061	56.68318
CHG	sensitive	45.7758	20.86598
CHH	sensitive	32.20361	22.45084

**Fig. 1:**
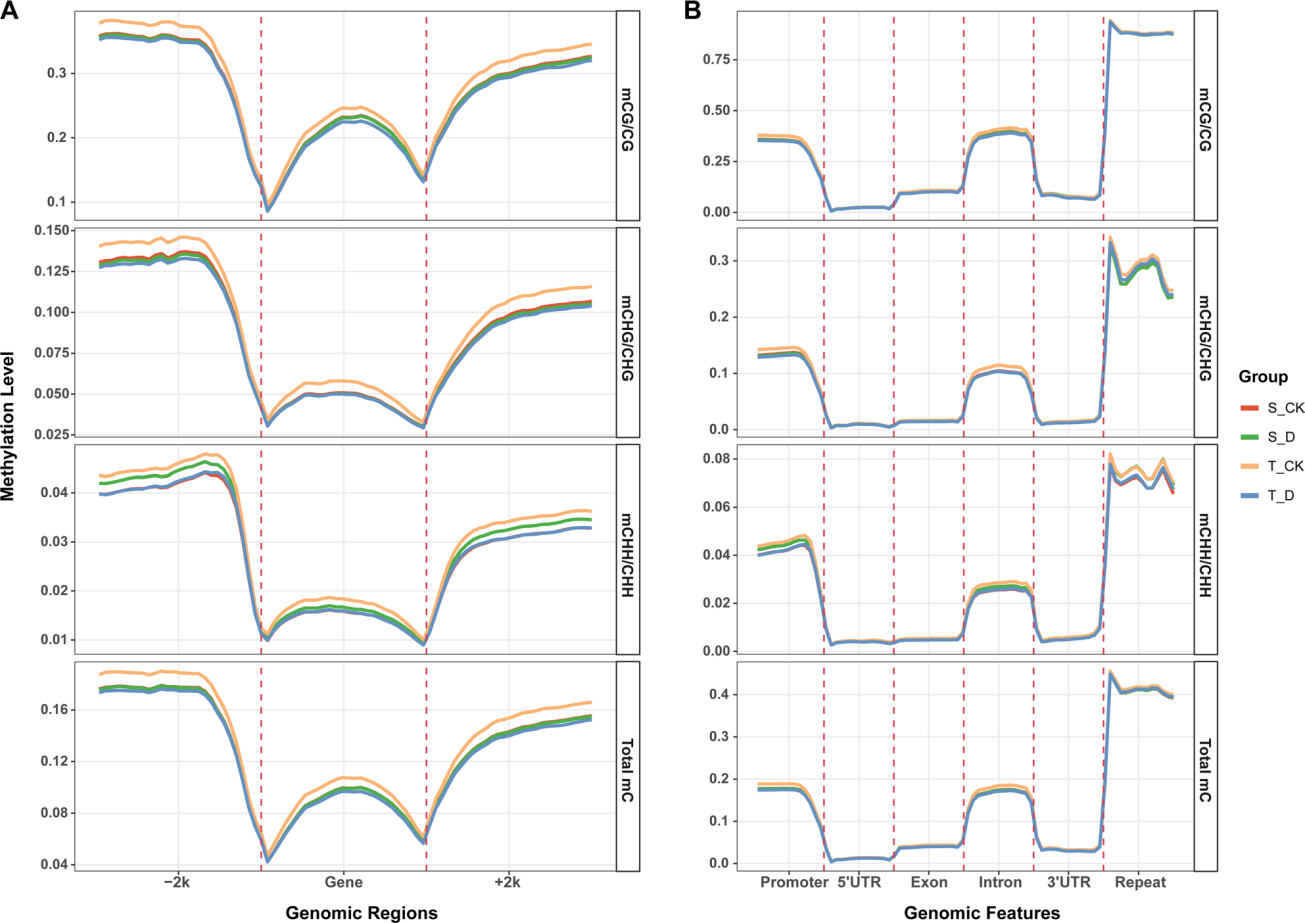
Global DNA methylation distribution patterns in Brassica napus. **A**. Distribution of CG, CHG, and CHH methylation levels across gene regions (2 kb upstream of TSS, gene body, 2 kb downstream of TES). Each region is divided into 50 bins. **B**. CG, CHG, and CHH methylation levels across different functional elements (including promoter, 5’ UTR, exon, intron, 3’ UTR, repeat). Each region is divided into 20 bins. For the selected 12 genotypes (6 drought-tolerant and 6 drought-sensitive), three biological replicates each for the watered (control) and drought-stress treatment were analyzed. Transcriptome and methylation sequencing were performed with two technical replicates per biological sample

Fine-scale analysis genomic features showed that CG methylation displayed the classic “U-shaped” profile along gene regions (Fig. 1 A), with higher levels in the upstream 2 kb (UP2000) and downstream 2 kb (DOWN2000) regions (~ 30%), compared to the gene body (19.6%). CHG methylation followed a similar but lower trend, while CHH methylation remained low (< 4%) throughout. Analysis of various functional elements further confirmed that CG methylation was highest across all annotated regions (Fig. 1B). For instance, promoter methylation was 32.6%, significantly exceeding CHG (12.1%) and CHH (4.0%). Notably, CG methylation in repeat regions reached 88.0%, highlighting its role in genome stability. Sample correlation demonstrated high reproducibility among biological replicates (Fig.S2).

This CG-centric conserved distribution aligns with fundamental plant epigenome architecture also were found in *Arabidopsis* and rice [28, 29]. Our results indicate similar static epigenomic landscapes between tolerant and sensitive materials before stress.

### Drought stress reshapes the DNA methylation landscape and reveals genotype-specific responses

We next investigated how drought stress reshapes this stable epigenomic architecture. Systematic identification of differentially methylated regions (DMRs) revealed pronounced DNA methylation reprogramming in response to drought stress in both drought-tolerant and drought-sensitive genotypes.

A global overview showed that CHH-DMRs predominated in both groups, constituting 69.6% (5552 of 7978) and 68.2% (4894 of 7174) of all significant DMRs in sensitive and tolerant materials, respectively (Table 2). This phenomenon indicates that asymmetric, non-CG methylation plays a central role in drought adaptation in rapeseed. Notably, sensitive materials had consistently exhibited a higher total number across all levels (conserved DMRs + 9.82%; significant DMRs + 11.21%; associated genes + 9.39%), indicating more extensive epigenomic reprogramming.

**Table 2:** Distribution characteristics of DMRs in *B. napus* materials with different drought resistances

Phenotype	Context	Conserved DMRs	Significant DMRs	Associated Genes
Tolerant	CG	1651	1362	1311
	CHG	1100	918	867
	CHH	5914	4894	4531
Sensitive	CG	1850	1515	1412
	CHG	1099	911	849
	CHH	6567	5552	5078
Common	CG	122	106	103
	CHG	27	22	22
	CHH	516	392	390

**Fig. 2:**
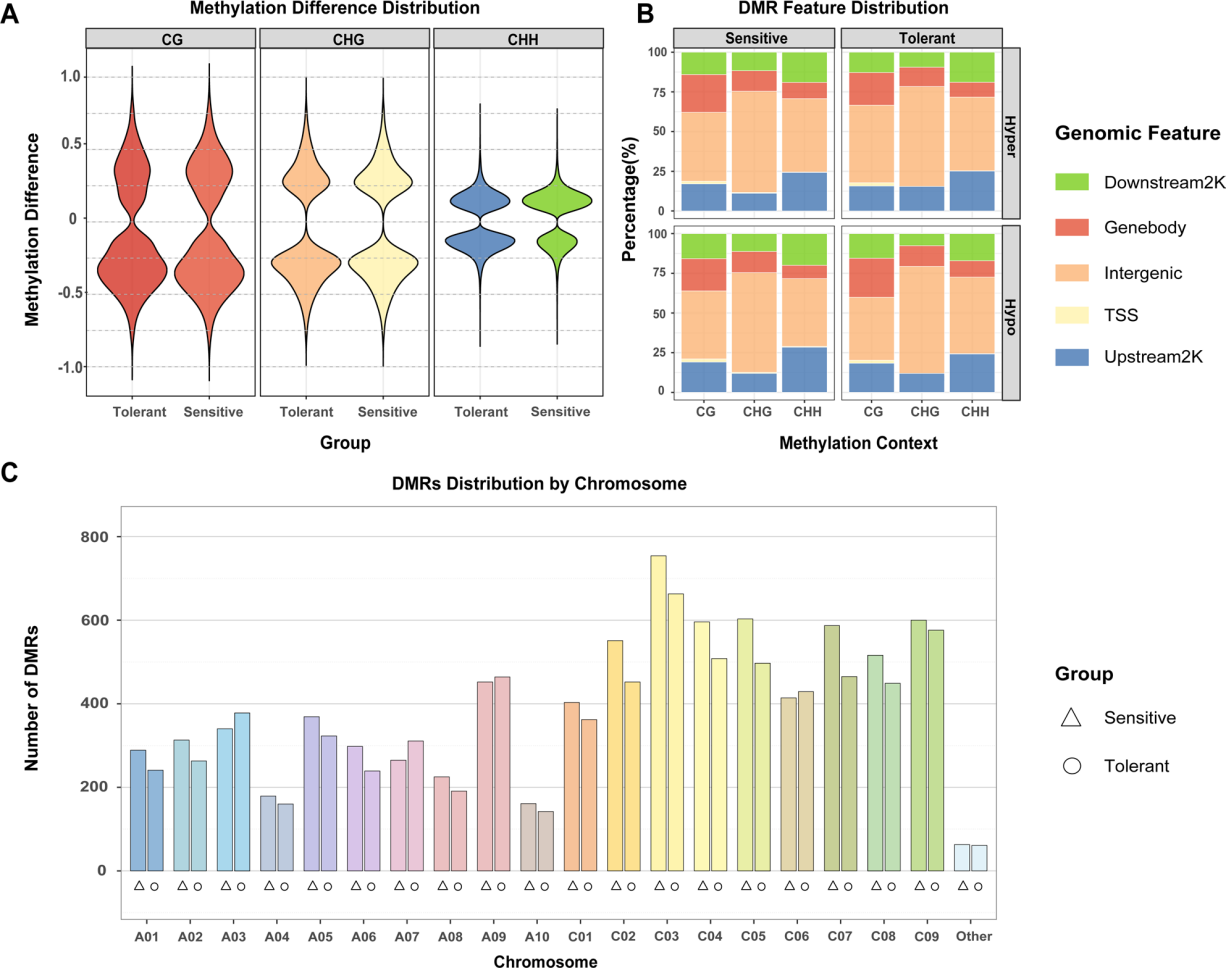
Characteristics of differentially methylated regions (DMRs) under drought stress. **A** Violin plots showing the distribution of methylation difference for significant DMRs in tolerant and sensitive groups across CG, CHG, and CHH sequence contexts. **B** Percentage distribution of DMRs from tolerant and sensitive groups, and common DMRs, across different genomic functional regions (e.g., upstream 2 kb, CDS, downstream 2 kb, intergenic), stratified by sequence context (CG, CHG, CHH). **C** Distribution density of DMRs from the tolerant group, sensitive group, and common DMRs across B. napus chromosomes, highlighting the dominance of the C subgenome and hotspot chromosome C03

In-depth analysis of the degree and direction of DMR methylation changes revealed two different epigenetic strategies (Fig. 2A). Sensitive materials exhibited a marked hyper-methylation bias, particularly in DMRs supported by multiple sample pairs. This bias towards hyper-methylation was consistently observed across DMRs supported by different numbers of sample pairs (Fig. S4), reinforcing the notion of a widespread epigenetic silence response in sensitive genotypes. In contrast, tolerant materials showed relatively balanced methylation changes with more concentrated change magnitudes. This divergence, most pronounced in the CHH context, highlights dynamic CHH reprogramming as a key differentiator [30].

Examination of DMR distribution across genomic features provided functional insights (Fig. 2B). The upstream 2 Kb gene region was the most enriched *cis-*regulatory element for DMRs, accounting for 25% of CHH-DMRs in both materials, respectively, indicating crucial promoter reprogramming. Simultaneously, CG-DMRs showed significant enrichment (14%) in coding sequence (CDS) regions, suggesting their potential involvement in stress regulation by post-transcriptional process. The genomic distribution of significant DMRs showed a clear subgenome preference (Fig. 2C). All eight chromosomes with the highest density belonged to the C subgenome with chromosome C03 emerging as a common hotspot in both groups. This predominant role of the C subgenome is consistent with prior studies highlighting its importance in the environmental adaptation of *Brassica* species [11, 28].

These functional distributions further revealed strategic differences between the two genotypes. Higher CG-DMRs in the upstream and downstream regulatory regions of the tolerant genotypes reflects its “precision-regulation” characteristic, whereas the epigenetic changes in sensitive genotypes more broadly affected gene body structural regions, consistent with “over-response” pattern.

### Functional divergence in methylome remodeling reflects adaptation strategies

To understand the functional implications of the observed methylome remodeling, we performed Gene Ontology (GO) enrichment analysis on genes associated with significant DMRs.

**Fig. 3:**
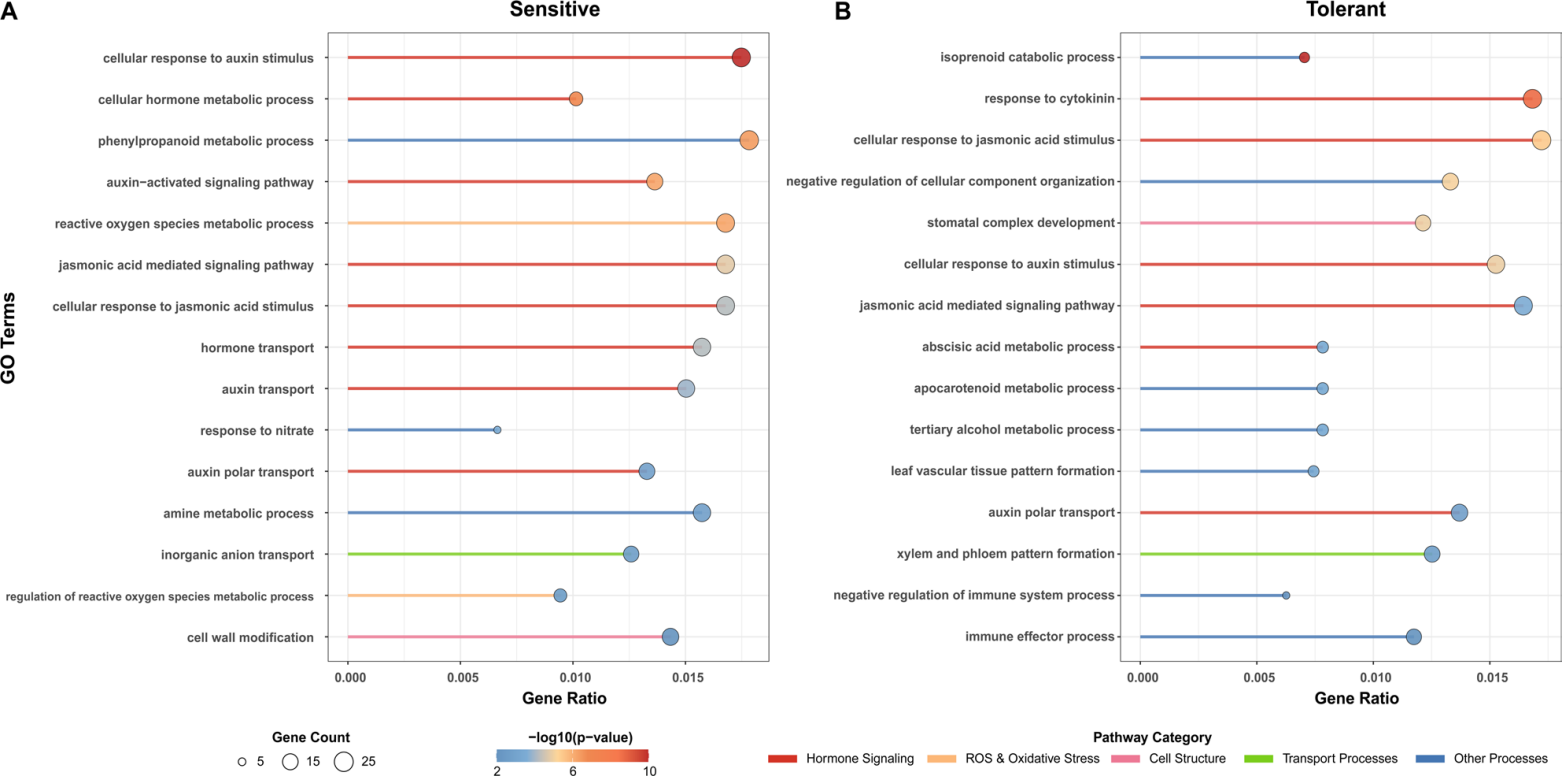
Functional enrichment analysis of DMR-associated genes reveals divergent adaptation strategies. **A**. Top 15 significantly enriched Biological Process (BP) pathways for DMR-associated genes in sensitive genotypes, highlighting auxin signaling and oxidative stress responses. **B**. Top 15 significantly enriched BP pathways for DMR-associated genes in tolerant genotypes, emphasizing developmental reconstruction and multi-hormone coordination. Dot size represents the number of enriched genes, and color represents the significance level of enrichment (-log₁₀(adjusted P-value))

In sensitive genotypes, 1,412 DMR-associated genes were significantly enriched in 165 biological pathways, indicating a typical rapid response profile (Fig. 3 A). Auxin signaling pathways were predominant, including key pathways “auxin-activated signaling pathway” “cellular response to auxin stimulus” and “auxin polar transport” [31], suggesting a substantial reliance on rapid hormone signaling activation. There also exhibited strong oxidative stress response characteristics. Strong oxidative stress responses were also evident, with significant enrichment of ROS metabolic processes [5]. Furthermore, phenylpropanoid metabolic process’s concurrent activation indicated structural defense mechanisms, such as cell wall reinforcement.

In contrast, 1,311 DMR-associated genes in tolerant genotypes were enriched in 229 pathways, demonstrating a more coordinated and diversified adaptation strategy (Fig. 3B). Hormonal regulation was more balanced, involving not only stress-related hormones like jasmonic acid and abscisic acid but also cytokinin response [32]. Notably, specific enrichment in developmental reconstruction process like “stomatal complex development” and “leaf vascular tissue pattern formation” indicated an optimization of water use efficiency through morphological adjustments. Meanwhile, the enrichment in “immune effector processes” reflected a better ability to balance defense and growth under stress [33].

The differentiation in functional enrichment patterns clearly corresponded to their epigenetic characteristics. The widespread, gene body-associated changes in sensitive genotypes align with a “rapid stress” strategy, while the precise, promoter-focused changes in tolerant genotypes support a “systemic adaptation” logic.

### Transcriptome analysis confirms and refines drought resistance strategies

To dissect the stress response mechanisms of these different genotypes at the transcriptional level, we conducted transcriptome sequencing on the same set of samples. High data quality assessment with ideal alignment efficiency, ensured robust analysis (Table S4, Table S5) [34].

**Fig. 4:**
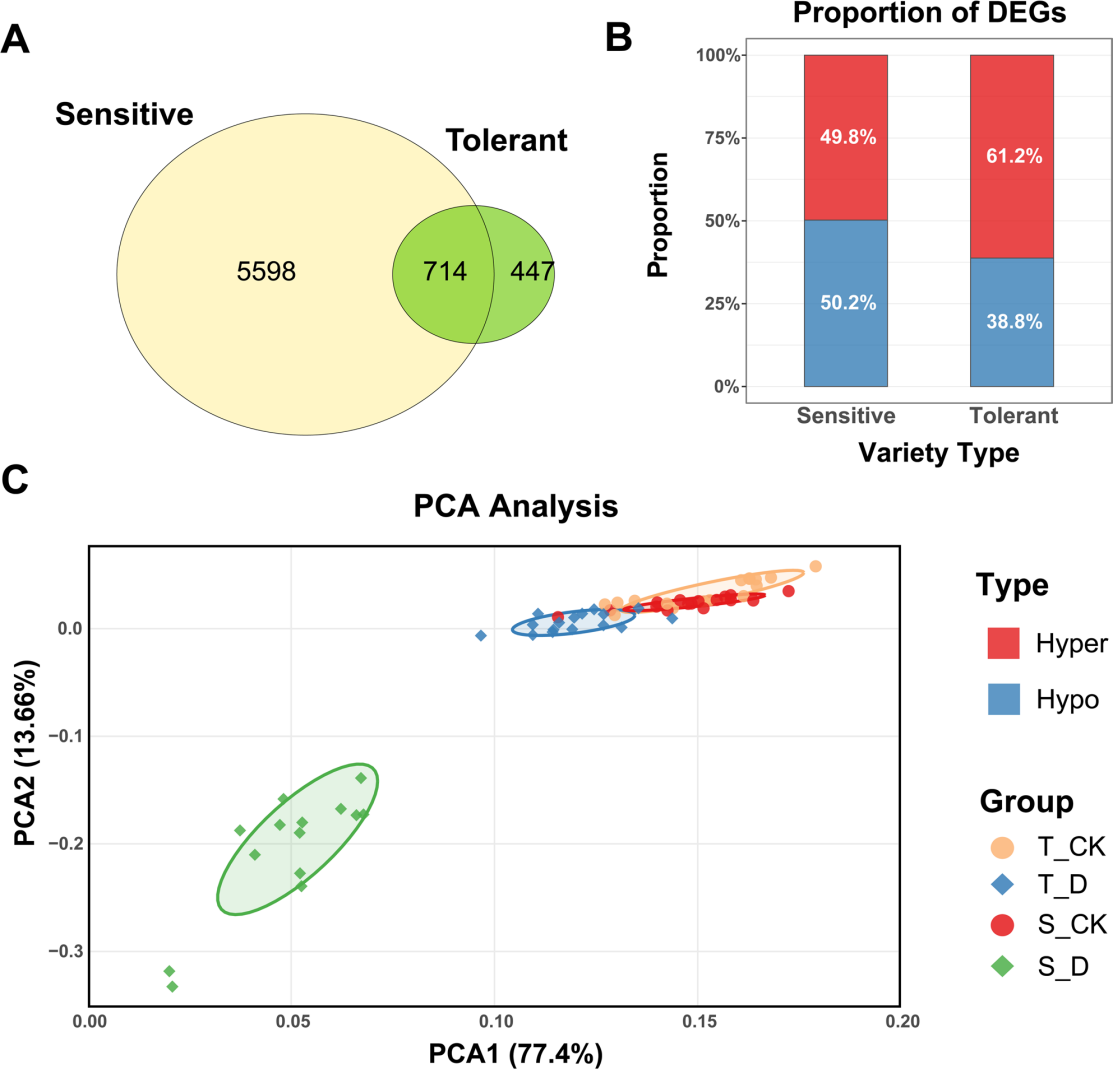
Transcriptome differential analysis under drought stress. **A**. Venn diagram showing the number of differentially expressed genes (DEGs) specifically in sensitive genotypes, tolerant genotypes, and those common to both. **B**. Proportion of up- and down-regulated DEGs in tolerant and sensitive genotypes. **C**. Principal component analysis (PCA) of transcriptome profiles for all samples under control and drought conditions, showing clear separation by genotype and treatment

Based on strict screening criteria, we identified a large number of drought-responsive differentially expressed genes (DEGs). Sensitive accessions had 6,312 significant DEGs, 5.4-fold more than tolerant accessions (1,161), indicating far greater transcriptomic perturbation (Fig. 4A). Venn diagram analysis further revealed strong genotype specificity, with only 714 common DEGs between the two groups; sensitive and tolerant genotypes possessed 5,598 and 447 specific DEGs, respectively.

Analysis of expression direction revealed fundamental regulatory differences (Fig. 4B). Tolerant genotypes showed a significant up-regulation preference (61.2% of DEGs), suggesting activation of tolerance or repair mechanisms. In contrast, the up- and down-regulated genes in sensitive genotypes were nearly balanced (49.8% vs. 50.2%), reflecting widespread and less coordinated transcriptional reprogramming. Principal component analysis (PCA) of the transcriptome data clearly separated samples by both genotype and treatment, validating the experimental design and the distinct transcriptional states (Fig. 4C). The distribution of all DEGs is displayed in volcano plots (Fig. S3).

**Fig. 5:**
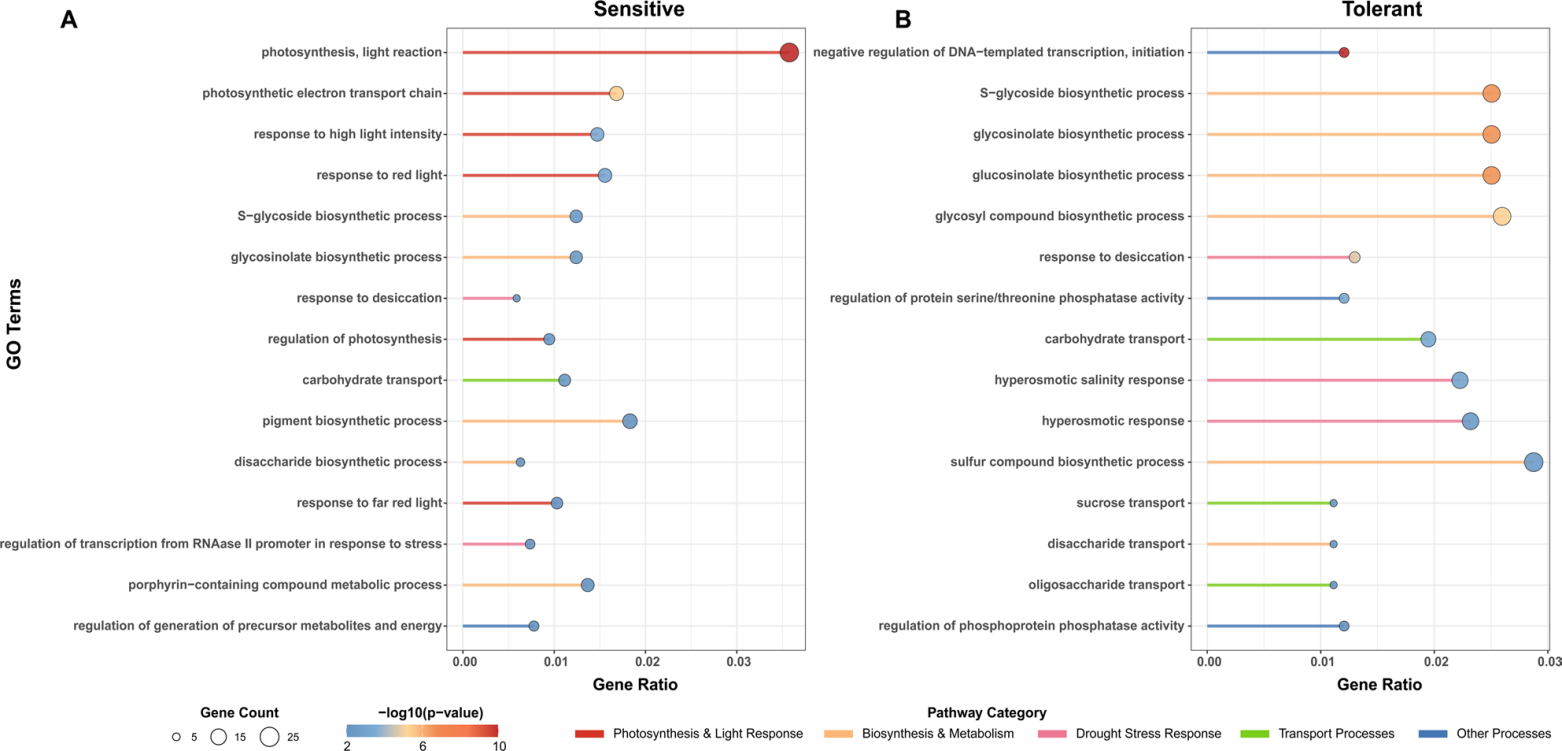
Functional characterization of transcriptional changes under drought stress. **A**. Top 15 significantly enriched Biological Process (BP) pathways for DEGs in sensitive genotypes, showing enrichment in photosynthesis and light response pathways. **B**. Top 15 significantly enriched BP pathways for DEGs in tolerant genotypes, highlighting carbohydrate transport and transcriptional regulation pathways. Dot size represents the number of enriched genes, and color represents the significance level of enrichment (-log₁₀(adjusted P-value))

GO enrichment analysis elucidated the biological significance of the transcriptional differences. In sensitive genotypes, DEGs were significantly enriched in photosynthesis and light response pathways (Fig. 5A), including “photosynthesis”, “light reaction”, and “response to high light intensity”. This “photosynthetic optimization-stress defense” profile suggests that sensitive plants attempt to maintain energy supply under drought, a strategy that may lead to energy depletion under persistent stress [35]. Conversely, DEGs in tolerant genotypes were enriched in a focused set of pathways related to resource management and systemic regulation (Fig. 5B). The most significant enrichments were in “carbohydrate transport” (e.g., sucrose transport) and processes related to transcriptional regulation. This “systemic regulation-metabolic reprogramming” profile indicates that tolerant plants achieve adaptation by prioritizing the transport and allocation of sugars and other osmoregulatory substances, a core strategy for efficient drought tolerance [36].

### Multi-omics integration identifies core drought response mechanisms

**Fig. 6:**
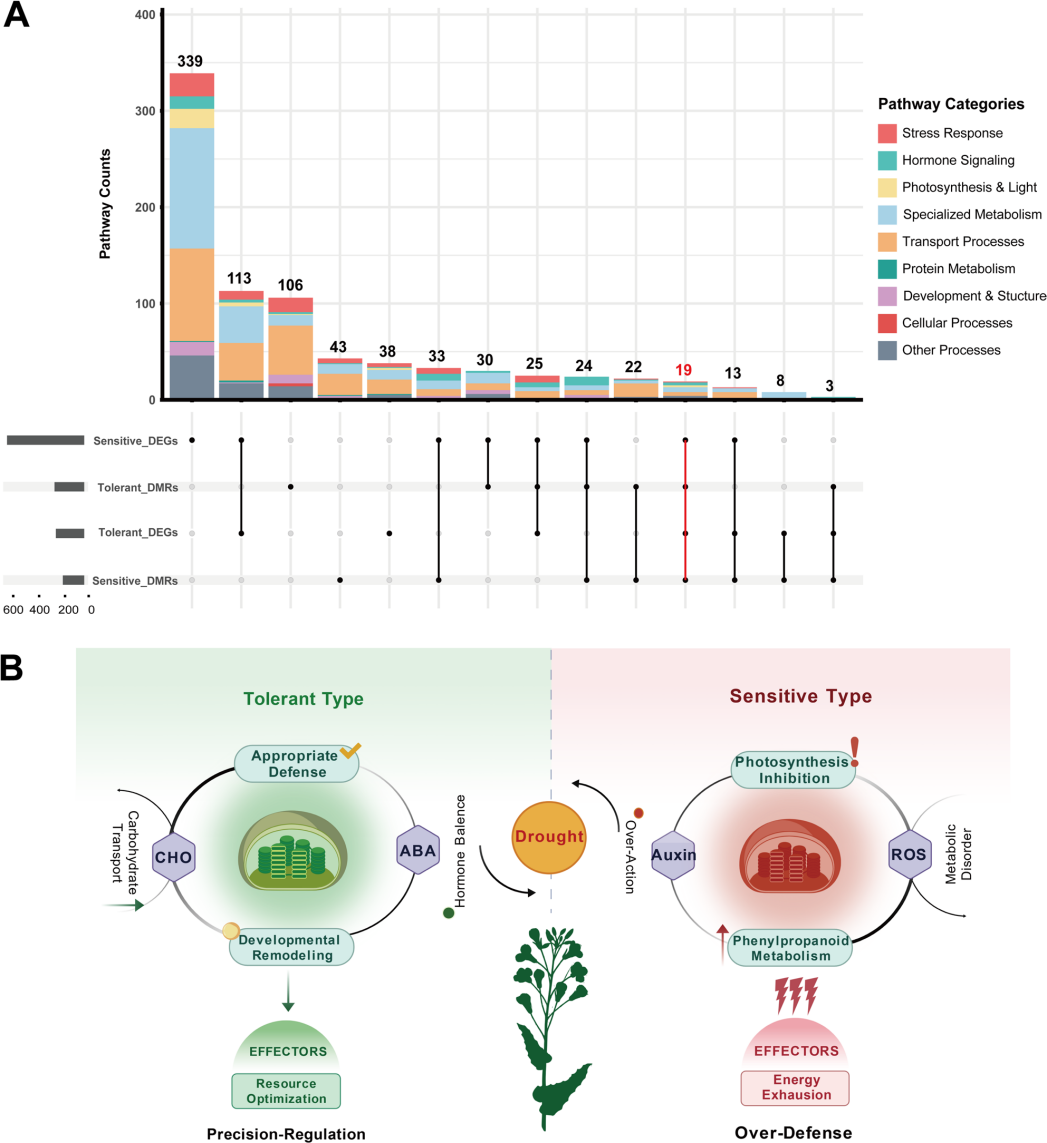
Multi-omics integration identifies core drought response mechanisms. **A**. UpSet plot showing the intersection of enriched pathways among the four datasets (sensitive DMR-associated genes, sensitive DEGs, tolerant DMR-associated genes, tolerant DEGs), identifying 19 core pathways enriched at both epigenetic and transcriptional levels. **B**. Cyclic molecular strategies for drought adaptation in rapeseed. The left panel illustrates the “precision-regulation” strategy of tolerant genotypes, characterized by JA/ABA balance, carbohydrate transport up-regulation, developmental reconstruction, and moderate defense, resulting in resource optimization. The right panel depicts the “over-defense” strategy of sensitive genotypes, featuring auxin over-activation, phenylpropanoid metabolism enhancement, ROS metabolic disorder, and photosynthesis inhibition, leading to energy exhaustion. Key genes associated with each strategy are indicated below each cycle. This diagram was created using BioGDP (Generic Diagramming Platform) and the silhouette of Brassica napus was obtained from PhyloPic

To systematically link the observed epigenetic reprogramming with transcriptional outcomes, we integrated the methylome and transcriptome datasets at the pathway level. An UpSet plot visualizing the intersections of enriched pathways across the four datasets revealed that while the sensitive genotype activated a vastly greater number of unique pathways (761 vs. 448 in tolerant), both genotypes shared a core module of 19 pathways that were significantly enriched at both the epigenetic (DMR-associated) and transcriptional (DEG) levels (Fig. 6A).

These 19 core pathways (Tables 3) represent fundamental molecular mechanisms co-regulated during drought adaptation and can be categorized into three key biological themes: (1) Structural Defense & Antioxidant, including phenylpropanoid metabolism which enhances cell wall integrity; (2) Signal Transduction & Homeostasis, such as jasmonic acid signaling and reactive oxygen species (ROS) metabolism crucial for stress perception; and (3) Environmental Perception & Resource Allocation, encompassing nitrate response, carbohydrate transport, and light signal perception, which reflect strategies for optimizing resource use.

**Table 3:** Functional classification of the 19 core pathways commonly enriched across the four omics datasets

Functional Category	Pathway Count	Representative Pathways
Phenylpropanoid Metabolism	2	phenylpropanoid metabolic process, phenylpropanoid biosynthetic process
Jasmonic Acid Signaling	2	jasmonic acid mediated signaling pathway, cellular response to jasmonic acid stimulus
Reactive Oxygen Species Metabolism	1	reactive oxygen species metabolic process
Nitrogen Metabolism	4	response to nitrate, amine metabolic process, inorganic anion transport, nitrate transport
Lipid Metabolism	4	lipid catabolic process, cellular lipid catabolic process, organic acid catabolic process, carboxylic acid catabolic process
Light Signal Perception	2	response to red light, photomorphogenesis
Carbohydrate Transport	1	carbohydrate transport
Cytokinin Biosynthesis	1	cytokinin biosynthetic process
Terpenoid Metabolism	2	isoprenoid catabolic process, sesquiterpenoid metabolic process

The divergent scale of pathway activation provided quantitative, multi-omics evidence for the two adaptation strategies. The sensitive genotype’s response was characterized by broad, less coordinated activation, with 415 genotype-specific pathways, nearly three times more than the tolerant genotype (144). Analysis of the functional distribution of these pathways (Fig. S5) further quantified this divergence: sensitive genotypes showed massive over-representation in specialized metabolism (+ 586%) and hormone signaling (+ 600%), hallmarks of an extensive “over-defense” effort. In contrast, the tolerant genotype’s response was highly focused, with transport processes constituting 45.8% of its activated pathways, epitomizing a “precision-regulation” strategy aimed at efficient resource allocation [37].

We synthesized these multi-omics findings into a conceptual model of cyclic molecular strategies (Fig. 6B). The model illustrates how drought stress triggers divergent regulatory cascades: in sensitive genotypes, an “over-defense” cycle involving auxin over-activation, enhanced phenylpropanoid metabolism, ROS disorder, and photosynthesis inhibition leads to energy exhaustion. Conversely, in tolerant genotypes, a “precision-regulation” cycle characterized by balanced JA/ABA signaling, upregulated carbohydrate transport, developmental reconstruction, and moderate defense results in resource optimization and sustained adaptation.

### Identification and characterization of epigenetic regulation hub genes

**Fig. 7:**
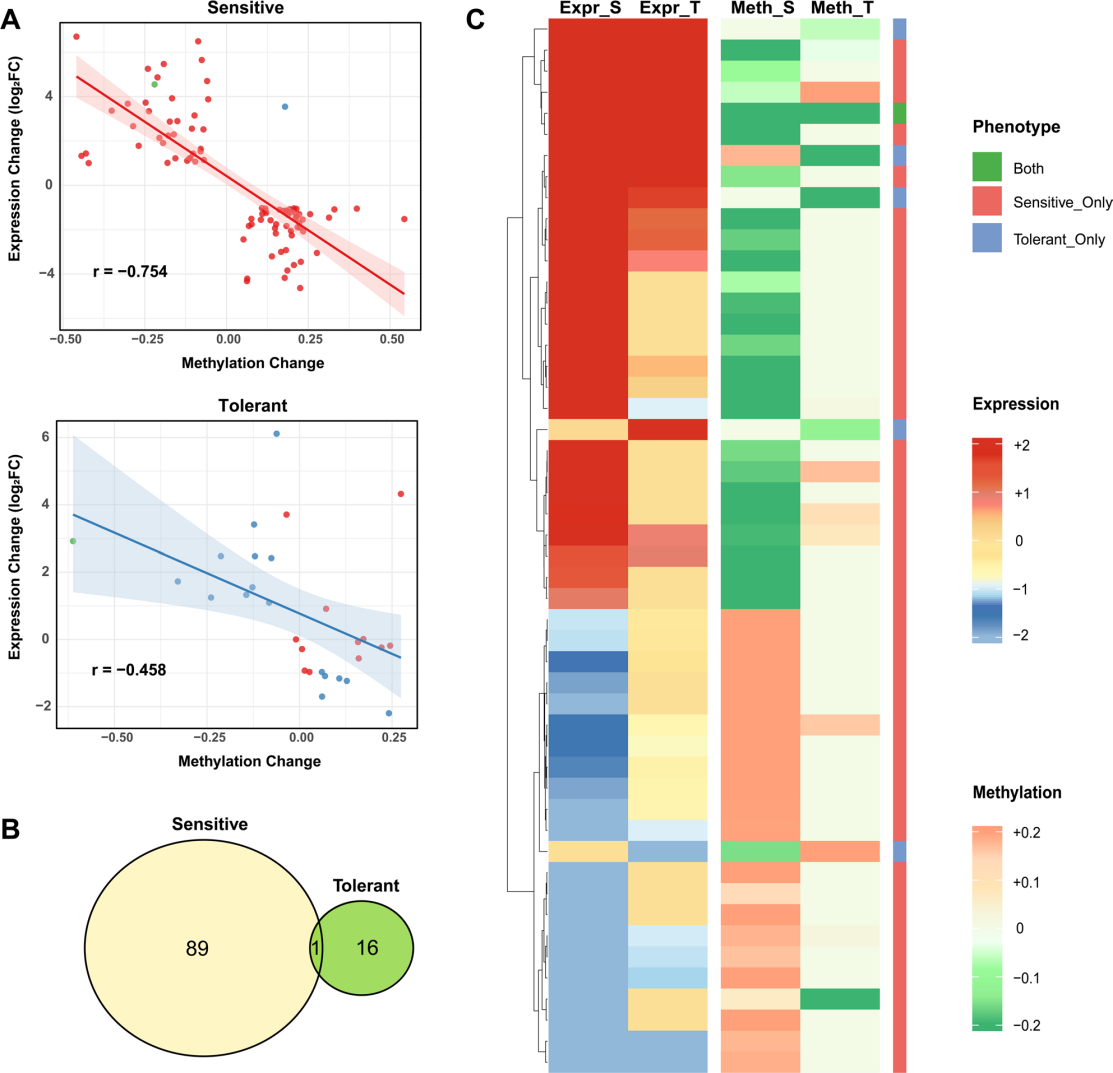
Identification and characterization of epigenetic regulation hub genes. **A**. Scatter plots showing methylation change versus expression change for genes conforming to the “methylation-expression” negative regulation pattern in sensitive and tolerant genotypes, annotated with Pearson correlation coefficient (r). **B**. Venn diagram showing the number of negatively regulated genes specifically identified in sensitive-type materials, tolerant-type materials, and their intersection. **C**. Heatmap showing expression levels (sensitive and tolerant groups) and methylation levels (sensitive and tolerant groups) of 15 high-priority epigenetically regulated candidate genes. Each row represents a gene; data are row-normalized (Z-score)

To delineate the direct molecular links between DNA methylation changes and transcriptional reprogramming, we searched genes exhibiting a “methylation-expression” negative regulation pattern. We found 106 high-confidence candidate genes, which showed strong genotype specificity: 89 were specific to the sensitive genotype, while only 16 were specific to the tolerant genotype, with 1 genes common to both (Fig. 7B). From these, we selected the top 15 high‑priority genes based on a comprehensive scoring system that integrates methylation–expression correlation strength (Table 4).

**Table 4:** High-priority epigenetically regulated candidate genes

Gene ID	Homolog	Phenotype Association
BnaA07G0032700ZS	ENA1	Both
BnaA02G0354700ZS	AFP3	Sensitive
BnaC04G0061900ZS	DTA2	Sensitive
BnaA02G0342800ZS	AITR1	Tolerant
BnaC02G0082400ZS	AT5G17680	Tolerant
BnaC07G0144800ZS	ERF12	Sensitive
BnaC02G0268300ZS	GER1	Sensitive
BnaC05G0254300ZS	LARP6a	Sensitive
BnaA02G0162000ZS	AT1G66880	Sensitive
BnaA09G0556900ZS	SYP73	Sensitive
BnaA01G0271000ZS	KT12	Sensitive
BnaC07G0311900ZS	AT3G28040	Sensitive
BnaC09G0286200ZS	CYP706A4	Sensitive
BnaA03G0417300ZS	COR413-PM2	Sensitive
BnaA09G0103500ZS	SCPL43	Sensitive

The strength of this negative correlation was significantly more pronounced in the sensitive genotype (the correlation coefficient, *r* = −0.754, *n* = 91) compared to the tolerant genotype (*r* = −0.458, *n* = 29) (Fig. 7A). This quantitative difference suggests that the widespread transcriptional dysregulation in sensitive plants is more directly and strongly coupled to extensive epigenetic changes, whereas the tolerant genotype’s precise regulation may involve additional, non-linear mechanisms. The genomic distribution of these negatively regulated genes also differed, with a higher proportion located in promoter regions in the sensitive genotype (Fig. S6). A heatmap of expression and methylation levels for the top 15 high-priority candidate genes clearly illustrates the pronounced co-variation in sensitive materials and more nuanced patterns in tolerant ones (Fig. 7C).

### Genome-wide and locus-specific regulation patterns under drought stress

**Fig. 8:**
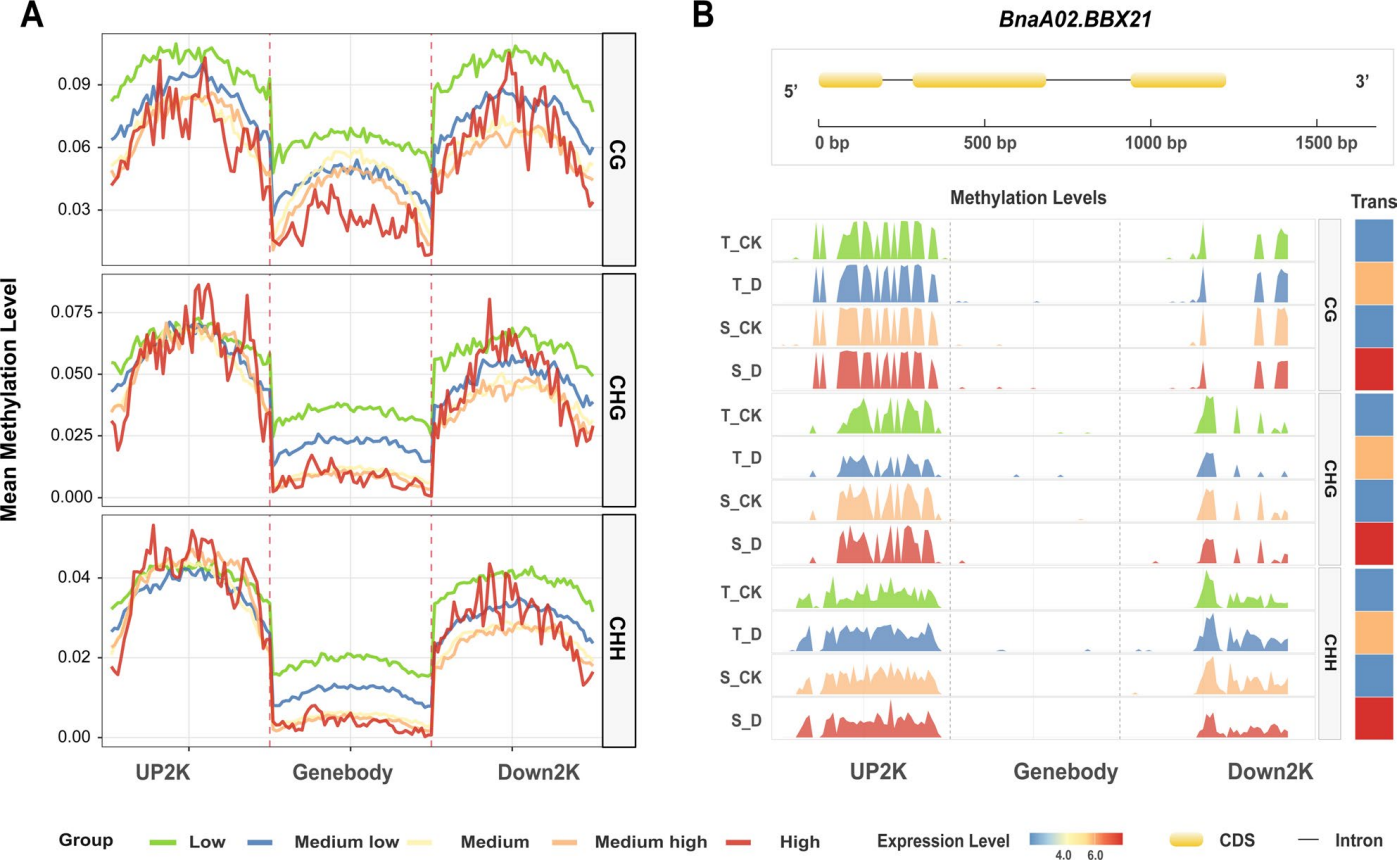
Genome-wide and locus-specific regulation patterns under drought stress. **A**. Relationship between DNA methylation (CG, CHG, CHH contexts) levels and gene expression levels under normal conditions. Genes were divided into six expression groups (none, low, medium low, medium, medium high, high) based on their average expression levels in control conditions across all accessions. The upstream 2 kb, gene body, and downstream 2 kb regions of each gene were each divided into 50 bins (150 bins in total). The average methylation level of each bin was calculated across all accessions under control conditions. **B**. Multi-omics view of the *BnBBX21* gene locus. The gene structure was drawn using GSDS (Gene Structure Display Server). DNA methylation patterns (CG, CHG, CHH) and expression levels are shown for sensitive and tolerant genotypes under control and drought conditions

To understand the baseline relationship between DNA methylation and gene expression, we analyzed their correlation under normal conditions. We categorized all genes into six expression groups and plotted the average methylation levels across their upstream, gene body, and downstream regions (Fig. 8A). This analysis revealed a conserved, negative correlation between CG/CHG methylation in promoter regions and gene expression levels. Interestingly, CHH methylation in the ~ 300 bp region upstream of the transcription start site showed a positive correlation with expression, a pattern consistent with regulatory “CHH islands” observed in other species.

Finally, to exemplify the locus-specific integration of multi-omics data, we examined key hub genes. *BnBBX21* (*BnaA02G0216200ZS*), encoding a B-box transcription factor implicated in ABA response and photosynthetic optimization, was specifically hypomethylated and upregulated in sensitive genotypes under drought [38] (Fig. 8B). Conversely, *BnTAT7* (*BnaC02G0171200ZS*), a tyrosine aminotransferase critical for vitamin E (antioxidant) biosynthesis, was specifically hypomethylated and strongly induced in tolerant genotypes (Fig. S8). We analyzed the coding and regulatory regions of the *BnBBX21* and *BnTAT7* using bcftools. The results revealed that the nucleotide sequences of these two genes were completely identical to the reference genome (ZS11) across the six drought-tolerant and six drought-sensitive cultivars we selected, with no single nucleotide polymorphisms (SNPs) or insertions/deletions (indels) detected. This finding suggests that the significant differences in ​transcriptional levels​ of these two genes between the drought-tolerant and sensitive cultivars are likely not attributable to variations in their nucleotide sequences. Instead, these differences may be caused by epigenetic modifications, such as DNA methylation.

## Discussion

Rapeseed (*Brassica napus* L.) is a globally important oil crop, and its productivity is increasingly challenged by abiotic stresses associated with climate change, particularly drought [11, 18]. Understanding the molecular mechanisms underlying drought tolerance is therefore essential for stabilizing yield and ensuring agricultural sustainability. In this study, we applied an integrated epigenomic and transcriptomic framework to systematically dissect drought responses in rapeseed genotypes with extreme and contrasting tolerance levels. We implemented several measures to ensure that the observed differences in methylation levels primarily reflected the effect of drought stress rather than confounding factors such as developmental stage or genetic background. First, plants were grown in controlled climate chambers to synchronize age and developmental progression, minimizing environmental influences on development. Second, the timing of drought application was strictly consistent, ensuring all plants were at a comparable physiological stage when stress began. Third, a standardized sampling protocol was followed to guarantee that all collected tissues were of identical age and physiological status at the moment of sampling. Finally, to reduce the genotypic bias, each group (drought-tolerant and drought-sensitive) included six genetically distinct genotypes. Differentially expressed genes or differentially methylated regions were only considered for the final set if they were identified in at least three genotypes within a group, allowing us to identify core response mechanisms representative of the group. By jointly analyzing whole-genome DNA methylation and gene expression dynamics, we provide a comprehensive, regulatory landscape that links stress-induced epigenetic remodeling to transcriptional reprogramming. A central finding of this work is the identification of two fundamentally distinct molecular response strategies to drought stress: a broad, intensive “over-defense” response in sensitive genotypes and a more restrained, targeted “precision-regulation” strategy in tolerant genotypes. These strategies are inferred from consistent patterns observed across methylome remodeling, transcriptional perturbation, and pathway-level integration, highlighting how different regulatory architectures may shape adaptive outcomes under drought stress.

### The central role of non-CG methylation in drought adaptation

Epigenetic regulation provided plants with a flexible and reversible mechanism to rapidly adjust gene activity in response to environmental stress [33, 39, 40]. Among epigenetic modifications, DNA methylation plays a particularly important role in modulating transcriptional responses and maintaining genome stability under adverse conditions [30]. Our results demonstrate that drought stress induces extensive DNA methylation reprogramming in *B. napus*, with non-CG methylation—especially in the CHH context—accounting for nearly 70% of all significant drought-responsive DMRs in both tolerant and sensitive genotypes (Table 2).

The predominance of CHH-DMRs underscores the importance of highly dynamic, RNA-directed DNA methylation (RdDM)-associated pathways in drought adaptation [41]. The enrichment of these CHH-DMRs in promoter regions (Fig. 2B) suggests a direct mechanism for modulating gene expression by influencing transcription factor binding or chromatin accessibility. This aligns with studies in other major crops like maize and rice, where stress-induced CHH methylation dynamics are intricately linked to targeted gene expression changes and represent a key rapid-response system [30, 42, 43]. However, our data reveal a critical nuance: differential drought resistance stems not merely from the involvement of CHH methylation, but from the nature of its reprogramming. The sensitive genotype exhibited a hyper-methylation bias and a general upward shift in CHH-DMR levels, indicative of a widespread, potentially indiscriminate silencing attempt. In contrast, the tolerant genotype showed balanced and more targeted changes. This suggests that the capacity for precise, context-appropriate CHH regulation, rather than its global activation, might underpin successful drought adaptation in *B. napus*.

### Subgenome asymmetry highlights the adaptive importance of the C subgenome

*Brassica napus* is an allopolyploid species comprising A and C subgenomes, which often exhibit functional divergence and regulatory asymmetry [14]. Increasing evidence indicates that such subgenome asymmetry plays a critical role in environmental adaptation [18]. Afsharyan et al. revealed a distinct bias in collective genomic structural-variation distribution toward the A subgenome, indicating it is more structurally fluid [44]. However, an asymmetrical selection pattern favoring the C subgenome was observed. This suggests a functional dichotomy: the C subgenome is under tighter selective constraint, likely preserving essential housekeeping functions, while the A subgenome acts as a laboratory for evolutionary experimentation and adaptation. Large-scale inversions were identified as critical drivers of diversification.

In this study, drought-induced DMRs showed a clear bias toward the C subgenome, with chromosome C03 emerging as a prominent hotspot of epigenetic remodeling in both genotypic groups (Fig. 2C). This C-subgenome dominance is consistent with previous reports demonstrating preferential stress-responsive gene expression, epigenetic plasticity, and structural variation within the C subgenome. One plausible explanation is that the C subgenome retains greater regulatory flexibility, enabling rapid epigenetic adjustment under environmental stress [20, 41, 44]. This asymmetric epigenetic responsiveness highlights the C subgenome as a particularly promising target for drought-resilient breeding and epigenetic marker development in rapeseed.

### Divergent molecular strategies: “over-defense” and “precision-regulation”

The study identifies and quantitatively delineates two fundamentally distinct drought adaptation strategies, linking the scale of epigenetic reprogramming directly to transcriptional outcome and resource allocation.

The sensitive genotype employs an energetically costly “over-defense” strategy, evidenced by a massive transcriptional response (6,312 DEGs) and the activation of a broad, uncoordinated network of pathways (Figs. 4A and 6A). Functionally, this strategy involved over-activation of auxin signaling, a hyperactive oxidative stress response, and attempts to maintain photosynthesis (Figs. 3A and 5A). This pattern reflects a “breadth-first” approach that mobilizes a vast array of defense mechanisms at a high energetic cost, consistent with the universal “growth-defense trade-off” [4]. Under sustained severe drought, this excessive investment, likely driven by widespread CHH hyper-methylation, may lead to metabolic exhaustion and inefficiency.

In contrast, the tolerant genotype may have deployed a “precision-regulation” strategy focused on economy and efficiency. It exhibited a tempered epigenomic response, a significantly smaller but focused transcriptional reprogrammation (1,161 DEGs), and a strong bias towards gene upregulation (61.2%) (Figs. 2A, 4A and B). Its functional signature was narrow and strategic, concentrated on enhancing carbohydrate transport and developmental processes for water use efficiency (Figs. 5B and 6B). By prioritizing the internal allocation of resources like sugars for osmotic adjustment, this strategy embodies a classic and efficient drought avoidance mechanism [36]. Therefore, successful drought tolerance in *B. napus* is determined less by the magnitude of defense gene activation and more by the precision and economy of internal resource allocation.

### Relationship between DNA methylation and gene expression

A major strength of this study lies in the integration of epigenomic and transcriptomic datasets, which enabled the identification of conserved regulatory modules underlying drought adaptation. Despite the pronounced differences in response magnitude between genotypes, both tolerant and sensitive genotypes shared a core set of 19 pathways that were significantly enriched at both methylome and transcriptome levels (Fig. 6A; Table 3). These pathways represent fundamental biological processes involved in structural defense, redox homeostasis, signal transduction, and resource allocation, forming a conserved regulatory backbone of drought response in *B. napus*. Reinforcing this finding, a recent multi-omics study on rapeseed flowers under drought also identified phenolic acid and flavonoid biosynthesis (key branches of phenylpropanoid metabolism) as central regulated pathways, highlighting the conserved role of these metabolic routes in drought adaptation across tissues [45].

To establish the direct molecular pathways, we performed a rigorous screen for gene pairs exhibiting a “methylation-expression” negative regulation pattern, identifying 106 high-confidence candidate genes. The significantly stronger negative correlation in sensitive genotypes (*r* = −0.754) compared to tolerant ones (*r* = −0.458) provides quantitative evidence that the sensitive genotype’s transcriptional dysregulation is more directly coupled to severe epigenetic perturbation (Fig. 7A).

The analysis successfully pinpointed 12 core hub genes at the convergence point of epigenetic control and pathway function (Table 5). These genes provide high-value targets for crop improvement, acting as the direct molecular bridges that define the two adaptation strategies.

**Table 5:** Hub genes at the intersection of core pathways and epigenetic regulation

Gene ID	Homolog	Phenotype Association
BnaA07G0371900ZS	PGM	Sensitive
BnaA02G0216200ZS	BBX21	Sensitive
BnaC07G0204600ZS	NRAMP4	Sensitive
BnaA07G0237900ZS	NIA1	Sensitive
BnaC03G0536200ZS	RGLG3	Sensitive
BnaA07G0075700ZS	IPT7	Sensitive
BnaC02G0179800ZS	SULTR1;1	Sensitive
BnaC04G0199700ZS	LOG1	Sensitive
BnaC03G0062800ZS	PAO1	Sensitive
BnaC04G0289100ZS	CSY2	Sensitive
BnaC02G0171200ZS	TAT7	Tolerant
BnaC08G0254400ZS	JMT	Tolerant

The contrasting strategies are best exemplified by two key hub genes: (1) *BnBBX21 (BnaA02G0216200ZS)*: A regulator that enhances drought tolerance by modulating ABA sensitivity and optimizing photosynthetic efficiency under water deficit [46]. *BnBBX21*’s low-methylation activation pattern is consistent with its rapid induction as part of the aggressive “over-defense” strategy. (2) *BnTAT7* (*BnaC02G0171200ZS*): The tyrosine aminotransferase *BnTAT7* was precisely activated in the tolerant genotype, epitomizing the “precision-regulation” strategy of reinforcing a targeted protective module. *BnTAT7* catalyzes a upstream step in the vitamin E (tocopherol) biosynthesis pathway [47], and the biosynthesis of this crucial lipid-soluble antioxidant is known to be upregulated during drought stress [48]. The remaining core hub genes further characterize this dichotomy, with defense-related genes like *GER1* and *CYP706A4* prominent in the sensitive network, while genes involved in signaling and homeostasis, such as LOG1 (a cytokinin activation enzyme) and JMT (a jasmonate signaling enzyme), emphasize the fine-tuning of physiological adjustments in the tolerant genotype.

The relationship between DNA methylation and gene expression is fundamentally determined by its genomic context. While promoter methylation, especially in CpG islands, typically silences genes by obstructing transcription factor binding or recruiting repressive complexes (e.g., MBDs and HDACs) [49], this is not a universal rule. The functional outcome critically depends on the location of the methylation [50]. For instance, methylation within the gene body is a common feature of actively transcribed genes and is associated with fine-tuning functions such as reducing transcriptional noise and guiding RNA splicing, indicating a role distinct from silencing. Furthermore, in specific contexts like tissue-specific genes with low-CpG-density promoters, methylation can be a prerequisite for activation by recruiting certain transcription factors essential for differentiation programs [51]. The sequence context itself is also pivotal, as methylation’s effect is primarily local, and its impact on transcription factors is highly variable, inhibiting some (e.g., CREB) while being inconsequential for others (e.g., Sp1) [52]. Therefore, DNA methylation operates as a nuanced regulatory mechanism where the genomic region and sequence context determine whether it serves to stably silence, fine-tune, or even activate transcription [53].

This context-dependent model provides the essential framework for interpreting specific experimental data, such as the weak genome-wide correlation (R² < 0.01) shown in Fig. S9. This overall weak correlation likely reflects the aggregation of opposing effects from different genomic regions. The observation that 131 genes (4.09%) exhibit a positive correlation between methylation and expression can be understood through this lens. This pattern is well-documented in plants, where gene body methylation is frequently associated with higher expression levels, a phenomenon observed in species like *Populus trichocarpa* [54], potentially ensuring efficient transcriptional elongation. Positive correlations have also been noted in specific upstream regions under stress conditions, and modifications like *N*^6^-methyladenine (6mA) near transcriptional start sites in rice further illustrate activating roles of methylation distinct from promoter repression [55, 56]. Consequently, the positive regulatory pattern observed in the subset of genes in Fig. S9 aligns with established biological mechanisms and should be discussed as a specific instance of gene body or regulatory element methylation, rather than an anomaly.

While this multi-omics approach robustly defines the molecular strategies underlying differential drought adaptation in *B. napus*, critical avenues for future investigation remain essential to translate these findings into applied molecular breeding. These areas for improvement represent high-priority research steps necessary for a complete mechanistic understanding. The current analysis captured the divergent strategies at a single, severe stress endpoint. This snapshot limits our ability to define the kinetic progression into the sensitive genotype’s unsustainable “over-defense” state. Future research should incorporate time-series multi-omics analysis across stress imposition and recovery phases. Time-resolved studies are vital for distinguishing transient epigenetic marks from stable, potentially heritable epialleles, which contribute to stress memory. Furthermore, while our integration provides statistically robust correlative evidence, translating this into confirmed mechanisms requires empirical functional genomics. The 12 core hub genes, especially *BnBBX21* and *BnTAT7*, must be prioritized for targeted functional validation. Using advanced genome editing tools like CRISPR/Cas9 to create targeted modifications is the essential next step to definitively confirm their direct, causal contribution to enhanced agronomic traits, such as yield stability under drought [57, 58].

Finally, the current robust regulatory model is based on bulk tissue analysis of DNA methylation, which inherently averages highly specific regulatory events across all cell types, potentially obscuring subtle, but crucial, localized dynamics. To achieve a complete hierarchical view, future studies should integrate orthogonal omics layers and single-cell/spatial resolution technologies. Incorporating small RNA sequencing and histone modification profiling (e.g., ChIP-seq) will clarify the upstream drivers of the observed CHH dynamics [41, 59]. More importantly, applying technologies like single-cell RNA-sequencing (scRNA-seq) would provide the necessary cellular resolution to clarify whether the tolerant genotype’s “precision-regulation” is a strong, highly targeted response restricted to specific cell types critical for water management. The successful application of single-nucleus RNA-seq to dissect cell-type-specific networks in *B. napus* flower buds demonstrates the feasibility and power of this approach in our crop system. Future studies focusing on drought-responsive tissues, such as roots and vasculature, will be crucial for revealing such precision regulation [60].

## Conclusions

This integrated epigenomic and transcriptional study elucidates the distinct drought adaptation mechanisms in *B. napus*. We proposed two contrasting strategies: a costly, broad “over-defense” response in sensitive genotypes, involving extensive CHH hypermethylation and massive transcriptional shifts that reduce efficiency, versus an economical “precision-regulation” strategy in tolerant genotypes, which maintains balanced regulation and prioritizes efficient carbon allocation via pathways like carbohydrate transport. Methodologically, multi-omics integration pinpointed 19 core pathways and 12 high-confidence hub genes that directly interface stress-induced epigenetic reprogramming with transcriptional responses. Notably, the C subgenome asymmetrically dominated the epigenetic response, offering a focused target for breeding. Moving forward, prioritizing temporal dynamics, functional validation of key genes (e.g., through gene editing), and expanded omics layers would enable translation of these insights into breeding strategies for drought-resilient rapeseed, enhancing agricultural sustainability under climate change.

## Methods

### Plant materials, drought stress treatment, and sampling

The experimental materials used in this study were selected from an association population consisting of 300 *B. napus* genetic accessions, which itself was selected from a rapeseed germplasm consisting of 991 accession from global origin (reserved at the Zhejiang Provincial Key Laboratory of Crop Germplasm Resources) that had undergone whole-genome resequencing [61]. To characterize the drought tolerance of rapeseed genotypes, we conducted a standardized drought stress experiment. Seedlings were cultivated for 15 days in a controlled environment chamber (16 h light/23°C, 8 h dark/21°C; light intensity 100–120 µmol/m²·s) until they developed 2–3 true leaves. The growth substrate was a sterilized 2:1 mixture of nutrient soil and vermiculite, placed in 16-cell trays. Drought stress was then applied by withholding water for 10 days. Sampling and phenotyping were initiated once over 30% of the genotypes exhibited visible wilting. Drought tolerance was assessed using the aboveground water content (WC1), calculated as WC1 = (Fresh Weight1 - Dry Weight1)/Fresh Weight1, which served as the core indicator. For the selected 12 genotypes, we analyzed three biological replicates. Transcriptome and methylation sequencing were performed with two technical replicates per biological sample. Detailed information for these genetic accessions is provided in Table S6.

The selected genetic accessions were cultivated in a controlled environment growth chamber. The experiment included a control group (normal watering) and a treatment group (drought stress induced by stopping watering). At 10 days after water withholding, when sensitive materials showed obvious wilting symptoms, the first true leaves of each genotype from both control and treatment groups were collected. All samples were immediately frozen in liquid nitrogen and stored at −80 °C until nucleic acid extraction.

### Library construction and sequencing

For transcriptomic and methylome analyses, samples were collected from 12 representative rapeseed accessions under both well-watered (control) and drought conditions. The samples comprised six extremely drought-tolerant and six extremely drought-sensitive genotypes. Total RNA for RNA sequencing was extracted from leaf tissues. In parallel, genomic DNA for whole-genome bisulfite sequencing was also extracted from the same 12 samples.

For transcriptome sequencing, mRNA with polyA tails was enriched using oligo(dT) beads, followed by fragmentation, reverse transcription, and strand-specific library construction protocols to prepare sequencing libraries. For methylome analysis, extracted genomic DNA was treated with bisulfite conversion, and then WGBS libraries were constructed. All libraries were subjected to paired-end sequencing (PE150) on the Illumina NovaSeq 6000 platform.

### Transcriptome data processing and differential expression analysis

Cutadapt (v4.4) was used for adapter and quality trimming of raw sequencing reads to obtain high-quality clean reads [62]. HISAT2 (v2.2.1) was then used to align the processed reads to the *B. napus* “Damor” reference genome [34]. StringTie (v2.2.1) was employed for transcript assembly, and ballgown (v2.34.0) was used to extract the gene expression count matrix [61].

Differential expression analysis was performed using the DESeq2 R package (v1.40.1) [35]. Low-expression genes with total counts below 10 across all samples were filtered out prior to analysis. The criteria for identifying differentially expressed genes (DEGs) were an adjusted P-value (P_adj_) < 0.05 and |log_2_FoldChange| > 1.

### WGBS data processing and differential methylation analysis

FastQC (v0.11.9) was used for raw data quality assessment, and Trim Galore (v0.6.7) was used for adapter trimming and quality control. Bismark (v0.24.0) software, invoking Bowtie2 (v2.5.1), was used to align the quality-controlled reads to the *B. napus* “ZS11” reference genome and extract whole-genome cytosine methylation information [25, 63].

Differentially methylated regions (DMRs) were identified using the DSS R package (v2.48.0) [19]. BEDTools (v2.30.0) was used to merge DMRs within the same phenotype group (tolerant or sensitive) with a maximum gap of 100 bp, defined as conserved DMRs [64]. The final screening thresholds for significant DMRs were set according to sequence context: CG context |Δmethylation level| > 20%; CHG context |Δmethylation level| > 15%; CHH context |Δmethylation level| > 25%.

### Gene annotation and functional enrichment analysis

BEDTools was used to annotate significant DMRs to genomic functional regions (e.g., promoter, gene body, intergenic). Gene functional enrichment analysis was performed using the online GO enrichment tool of the BnIR database for DEGs and DMR-associated genes separately, using a corrected P-value (P_adj_) < 0.01 as the threshold for significant enrichment.

### Multi-omics integration analysis

Homologous gene correspondence from the BnIR database (https://yanglab.hzau.edu.cn/BnIR/) was used to uniformly map transcriptome data based on the “Damor” genome to the “ZS11” genome annotation system for integrated analysis.

To screen for high-confidence epigenetically regulated candidate genes, we took the intersection of DMR-associated genes located within gene bodies or their upstream/downstream 2 kb regions and DEGs, and filtered for gene pairs conforming to the “methylation-expression” negative regulation pattern (screening criteria: |methylation_diff| > 0.05). The strength of epigenetic regulation was assessed by calculating the Pearson correlation coefficient between methylation level changes and expression level changes for these genes. We established a comprehensive priority scoring system (Priority_score = (Expression change magnitude) × 1 + (Methylation change magnitude) × 5 + (Phenotype coverage) × 10 + (Data completeness) × 5 + log(DMR count) × 2) to rank candidate genes. Finally, core hub genes were identified by intersecting the aforementioned candidate genes with the genes corresponding to the 19 core pathways.

### Statistical analysis and visualization methods

All statistical analyses and data visualizations were performed in R (version 4.2.0) using the following packages: ggplot2 (v3.4.3), pheatmap (v1.0.12), grid (v4.2.0), dplyr (v1.1.0), tidyr (v1.3.0), data.table (v1.14.8), and patchwork (v1.1.2). Correlation analyses were performed using Pearson correlation coefficients. Multiple testing correction was applied using the Benjamini-Hochberg method.

For the analysis of genome-wide methylation-expression relationships under normal conditions (Fig. 8A and C), genes were divided into six expression groups based on their average expression levels in control conditions across all accessions. For each gene, the upstream 2 kb, gene body, and downstream 2 kb regions were each divided into 50 bins (150 bins in total). The average methylation level of each bin was calculated across all accessions under control conditions.

The molecular strategies diagram (Fig. 6B) was created using BioGDP (Generic Diagramming Platform, https://www.biogdp.com). The silhouette of Brassica *napus* was obtained from PhyloPic (http://phylopic.org) [65]. The gene structure diagrams for *BnBBX21* (Fig. 8D) and *BnTAT7* (Fig. S8) were generated using GSDS (Gene Structure Display Server, http://gsds.cbi.pku.edu.cn).UpSet plot (Fig. 6A) was generated using the UpSetR (v1.4.0) package in R to visualize the intersections of enriched pathways across the four datasets (sensitive DMR-associated genes, sensitive DEGs, tolerant DMR-associated genes, tolerant DEGs).

## Supplementary Information

Additional file 1: Figure S1. Pie chart showing the average methylation proportion across 24 samples in three sequence contexts (CG, CHG, CHH)

Additional file 2: Figure S2. Heatmap of genome-wide methylation level correlations among 24 samples in CG, CHG, and CHH contexts, calculated based on 2 kb windows (bins)

Additional file 3: Figure S3. Volcano plots of DEGs in the sensitive group and tolerant group under drought stress

Additional file 4: Figure S4. Distribution of hyper- and hypo-methylated DMRs supported by different numbers of sample pairs (3-6 pairs) in tolerant and sensitive groups

Additional file 5: Figure S5. Ridge plots showing the distribution of methylation difference for all significant DMRs in tolerant and sensitive groups. Dashed lines indicate the median of each distribution

Additional file 6: Figure S6. Distribution proportion of all 106 negatively regulated genes across promoters, gene bodies, and intergenic regions

Additional file 7: Figure S7. Clustering analysis of genome-wide DNA methylation profiles based on CG, CHG, and CHH methylation levels across all samples

Additional file 8: Figure S8. Multi-omics view of the *BnTAT7* gene locus. The gene structure was drawn using GSDS (Gene Structure Display Server). DNA methylation patterns (CG, CHG, CHH) and expression levels are shown for sensitive and tolerant genotypes under control and drought conditions

Additional file 9: Figure S9. DNA methylation and gene expression relationships for differentially modified and expressed genes under drought stress. Correlation between DNA methylation changes (ΔMethylation > |0.05|) and gene expression changes (log_2_ fold change, padj < 0.05) in sensitive and tolerant genotypes. Analysis includes 3,206 genes that showed significant changes in both methylation and expression. Genes are categorized by their regulation pattern: negative regulation (blue, expression and methylation changes in opposite directions), positive regulation (red, expression and methylation changes in the same direction), and other genes (gray, no coordinated significant changes). Solid regression lines indicate the linear relationship specifically for negative regulation genes in each genotype. The top-left annotation displays the coefficient of determination (R²) for all genes combined, highlighting the weak genome-wide association

Additional file 10: Table S1-S6

## Data Availability

The bisulfite sequencing and RNA sequencing data generated in this study have been deposited in the China National Center for Bioinformation (CNCB-NGDC) under BioProject accession number PRJCA053295 (https://ngdc.cncb.ac.cn/bioproject/browse/PRJCA053295).

## Abbreviations

ABA: Abscisic acid
BP: Biological process
CDS: Coding sequence
DEG: Differentially expressed gene
DMR: Differentially methylated region
FPKM: Fragments per kilobase of transcript per million mapped reads
GO: Gene ontology
GWAS: Genome-wide association study
JA: Jasmonic acid
ROS: Reactive oxygen species
TSS: Transcription start site
TES: Transcription end site
UTR: Untranslated region
WGBS: Whole-genome bisulfite sequencing
