# Supplementary figures and images for: Integrated epigenomic and transcriptional profiling reveals genotype-specific adaptive reprogramming to drought stress in Brassica napus
Source: BMC Plant Biol. 2026 Mar 2;26:625. doi: 10.1186/s12870-026-08405-0 (PMC13059363; doi:10.1186/s12870-026-08405-0)

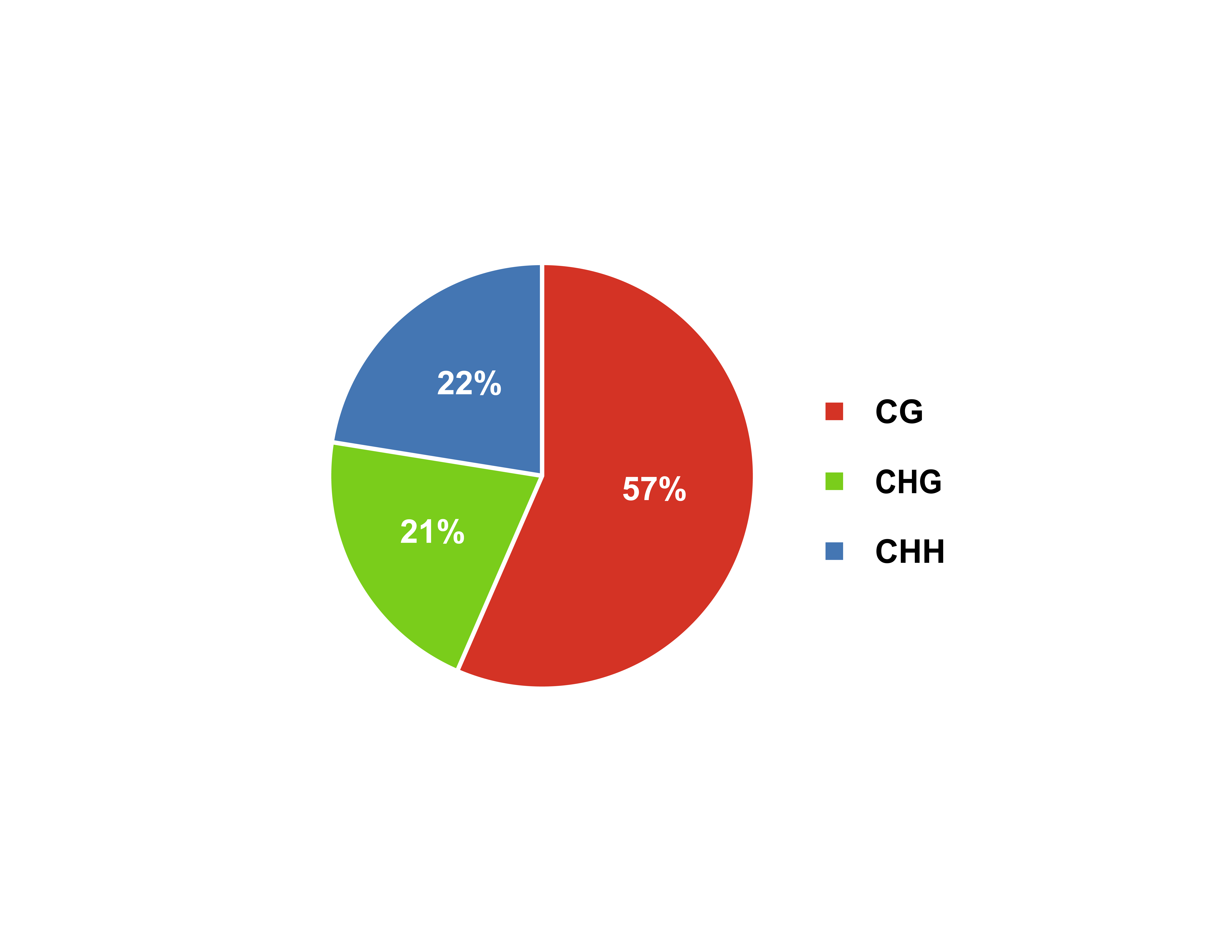

Supplement: Supplementary file 1 — Additional file 1: Figure S1. Pie chart showing the average methylation proportion across 24 samples in three sequence contexts (CG, CHG, CHH) [file 12870_2026_8405_MOESM1_ESM.tif]

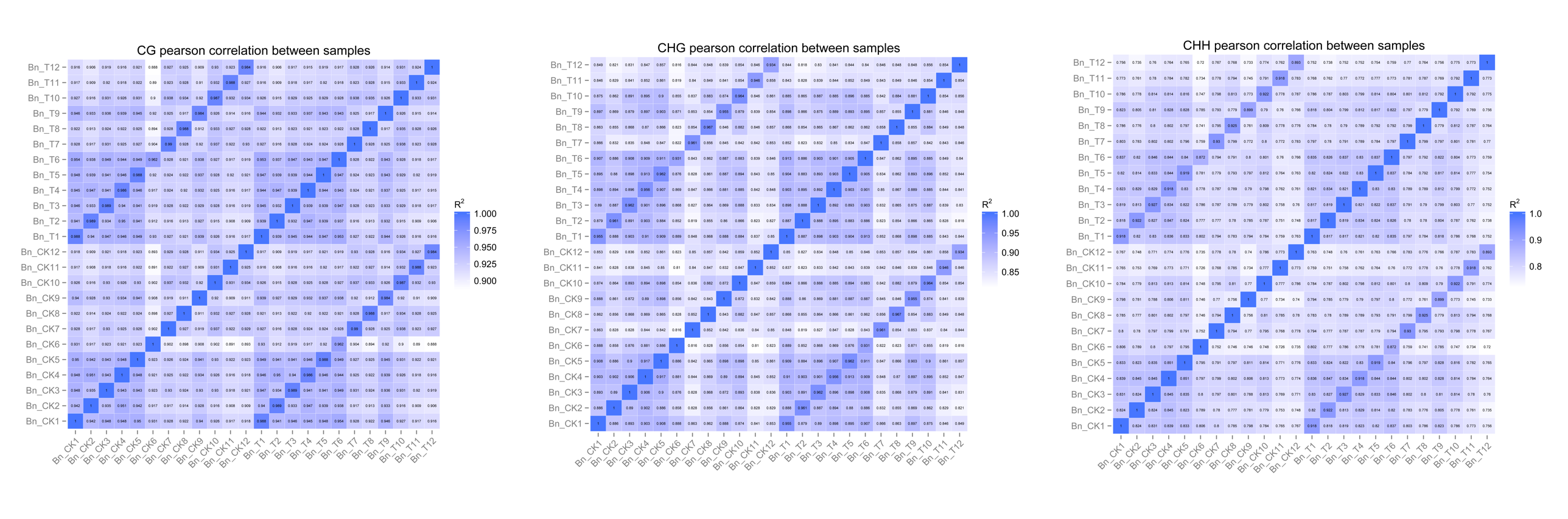

Supplement: Supplementary file 2 — Additional file 2: Figure S2. Heatmap of genome-wide methylation level correlations among 24 samples in CG, CHG, and CHH contexts, calculated based on 2 kb windows (bins) [file 12870_2026_8405_MOESM2_ESM.tif]

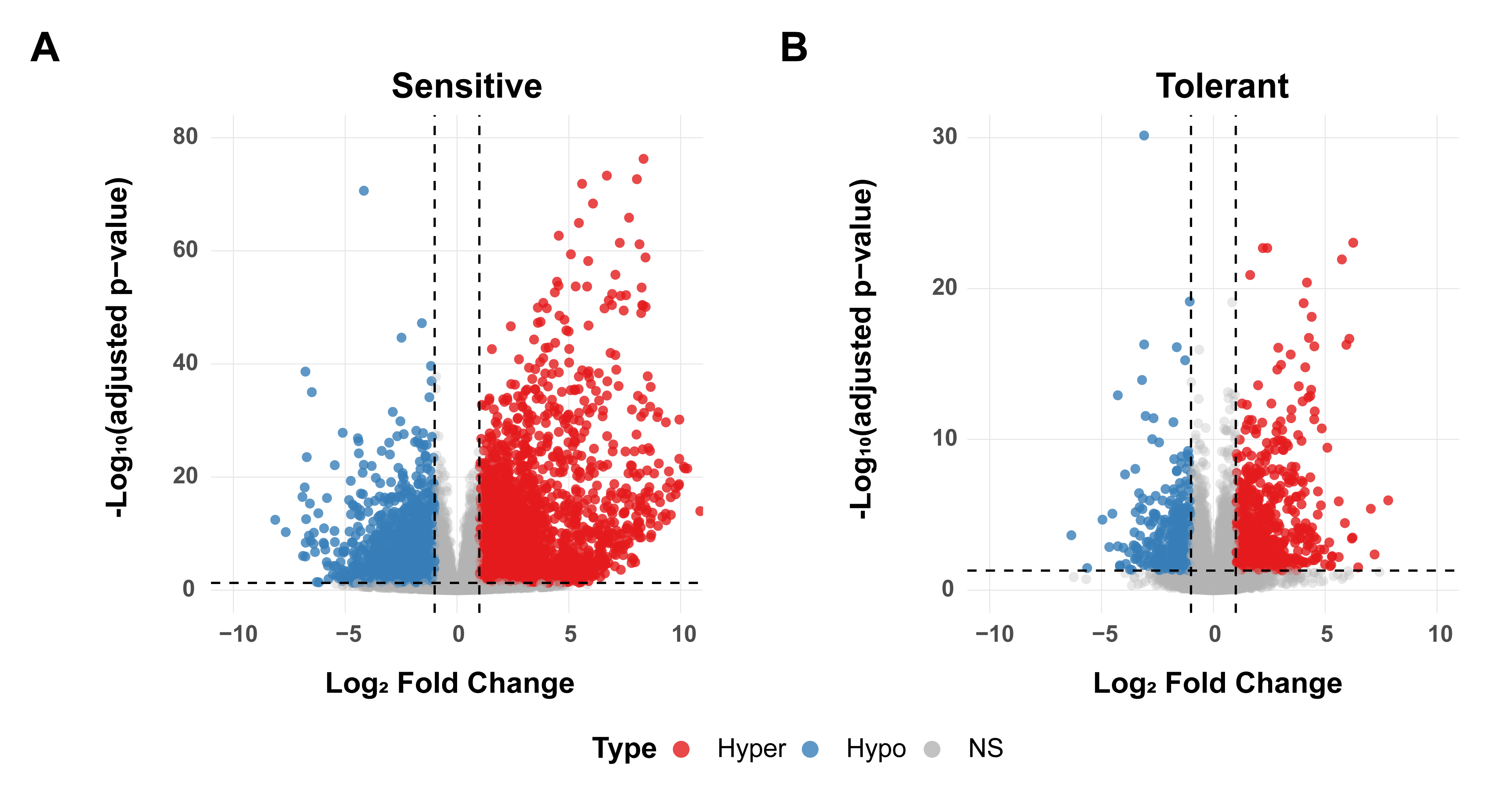

Supplement: Supplementary file 3 — Additional file 3: Figure S3. Volcano plots of DEGs in the sensitive group and tolerant group under drought stress [file 12870_2026_8405_MOESM3_ESM.tif]

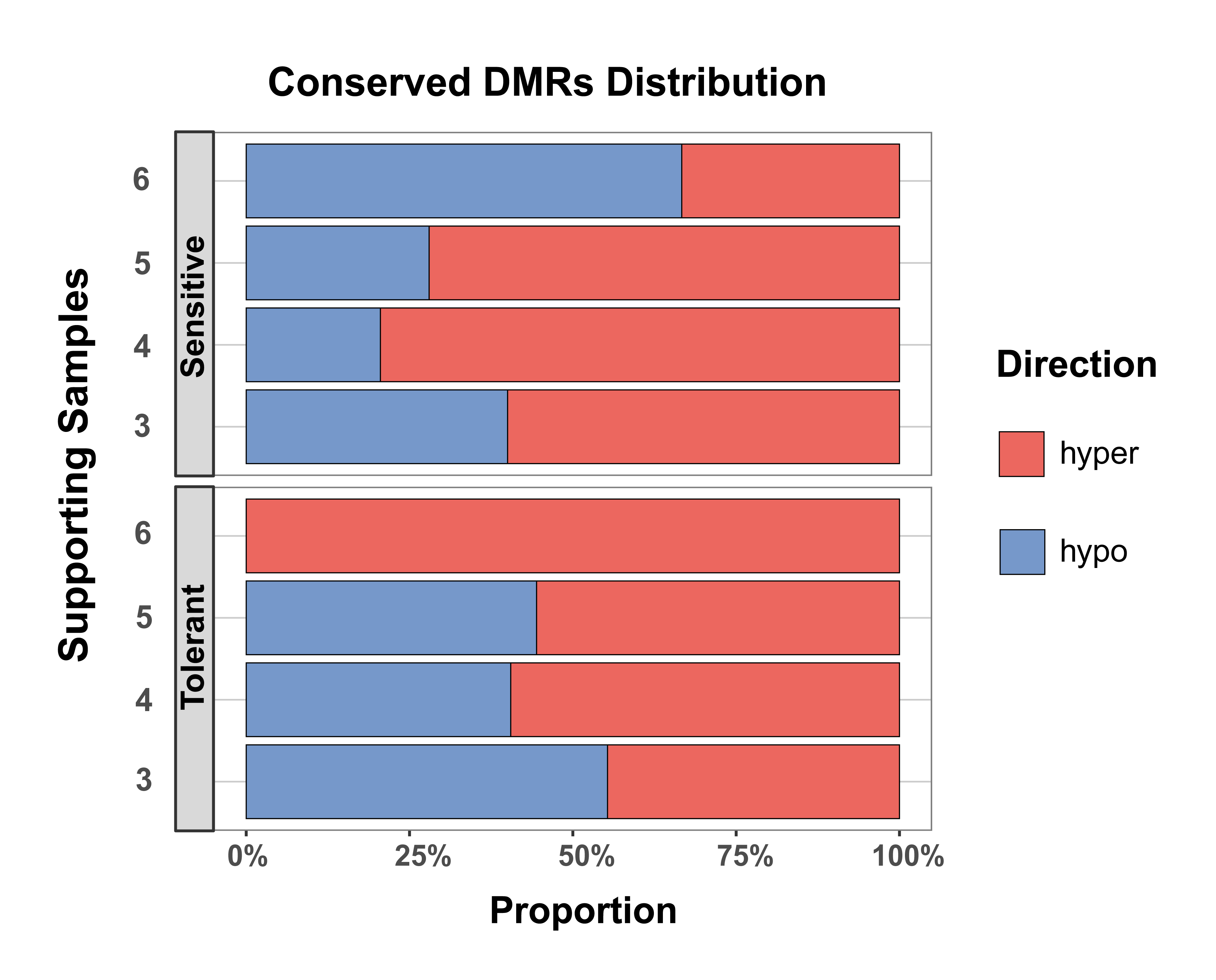

Supplement: Supplementary file 4 — Additional file 4: Figure S4. Distribution of hyper- and hypo-methylated DMRs supported by different numbers of sample pairs (3-6 pairs) in tolerant and sensitive groups [file 12870_2026_8405_MOESM4_ESM.tif]

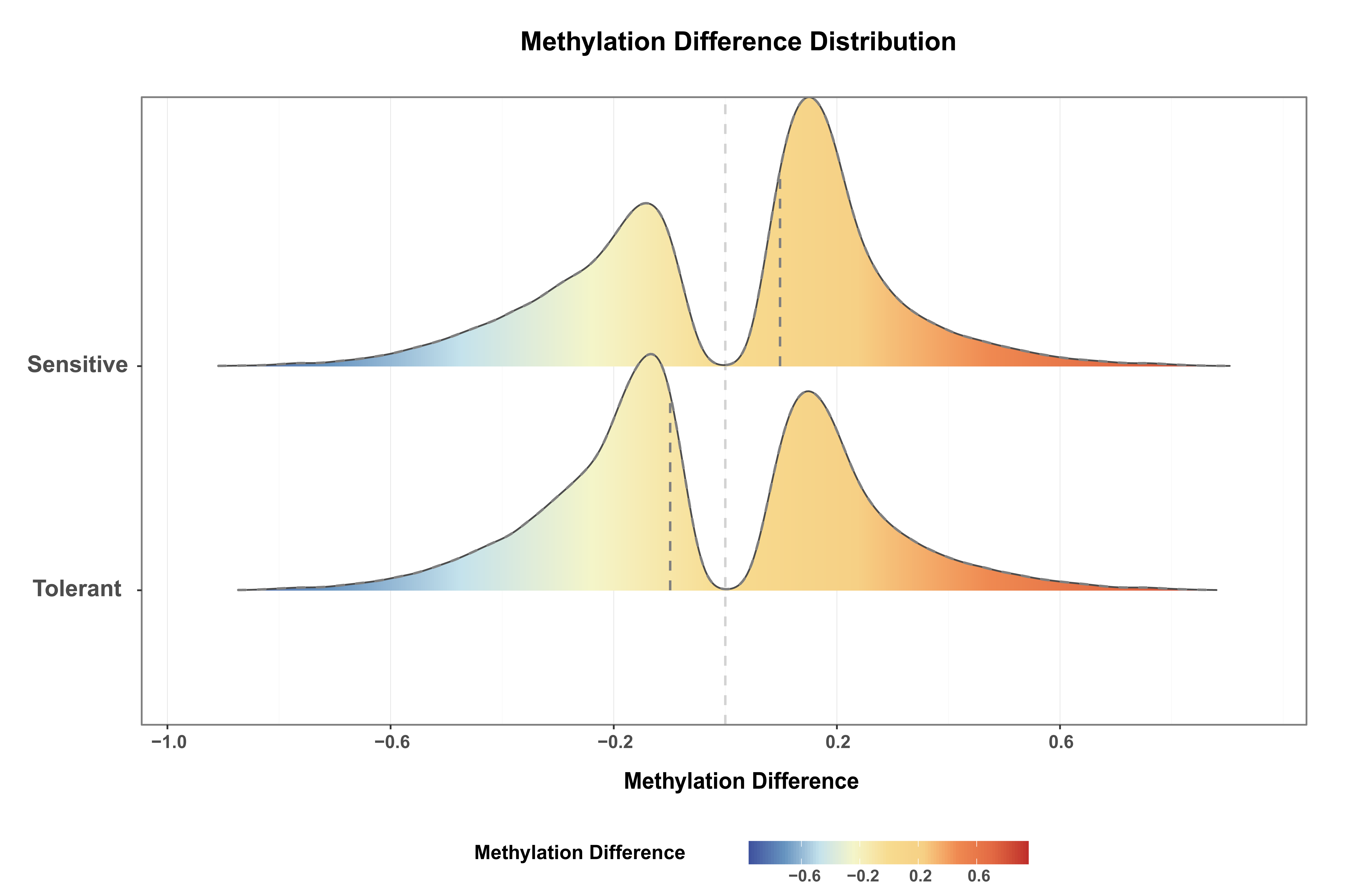

Supplement: Supplementary file 5 — Additional file 5: Figure S5. Ridge plots showing the distribution of methylation difference for all significant DMRs in tolerant and sensitive groups. Dashed lines indicate the median of each distribution [file 12870_2026_8405_MOESM5_ESM.tif]

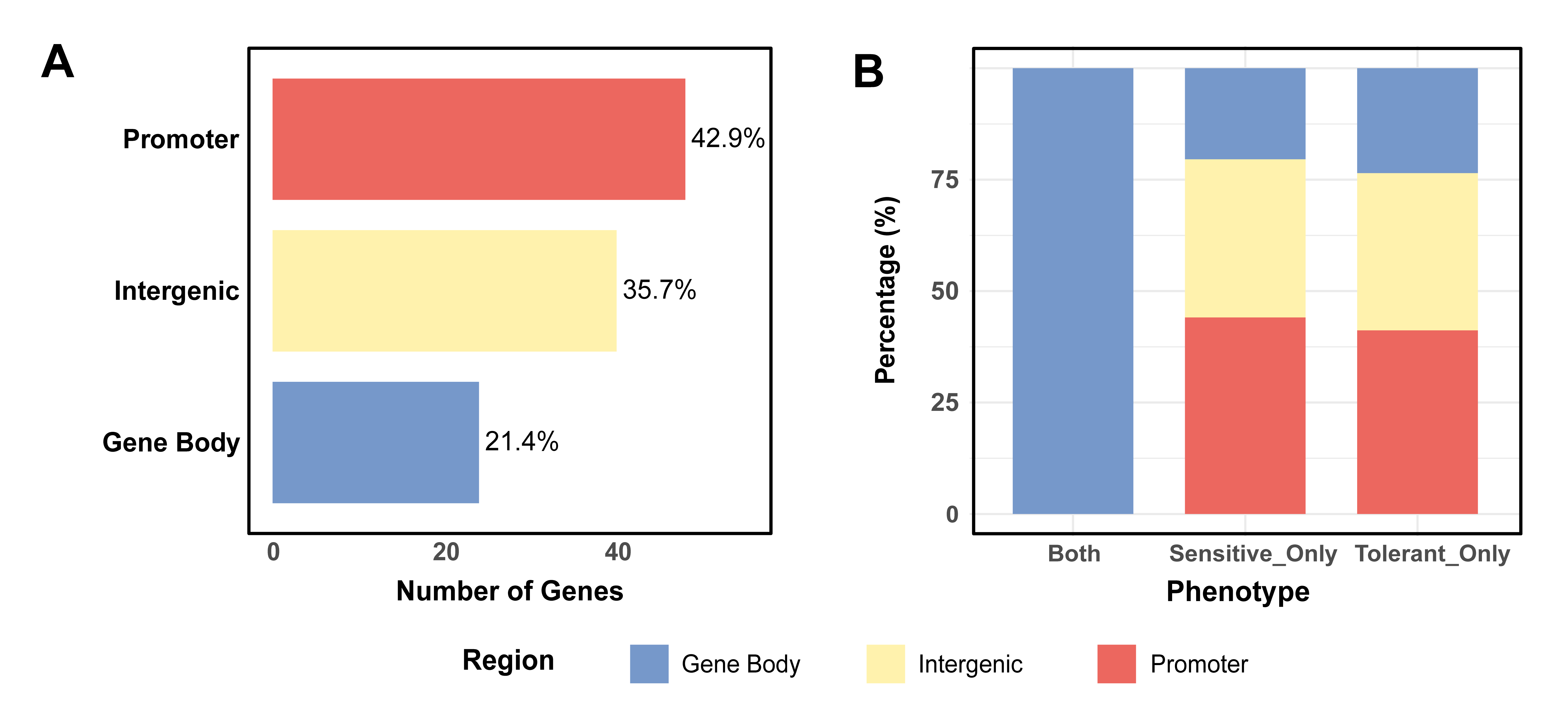

Supplement: Supplementary file 6 — Additional file 6: Figure S6. Distribution proportion of all 106 negatively regulated genes across promoters, gene bodies, and intergenic regions [file 12870_2026_8405_MOESM6_ESM.tif]

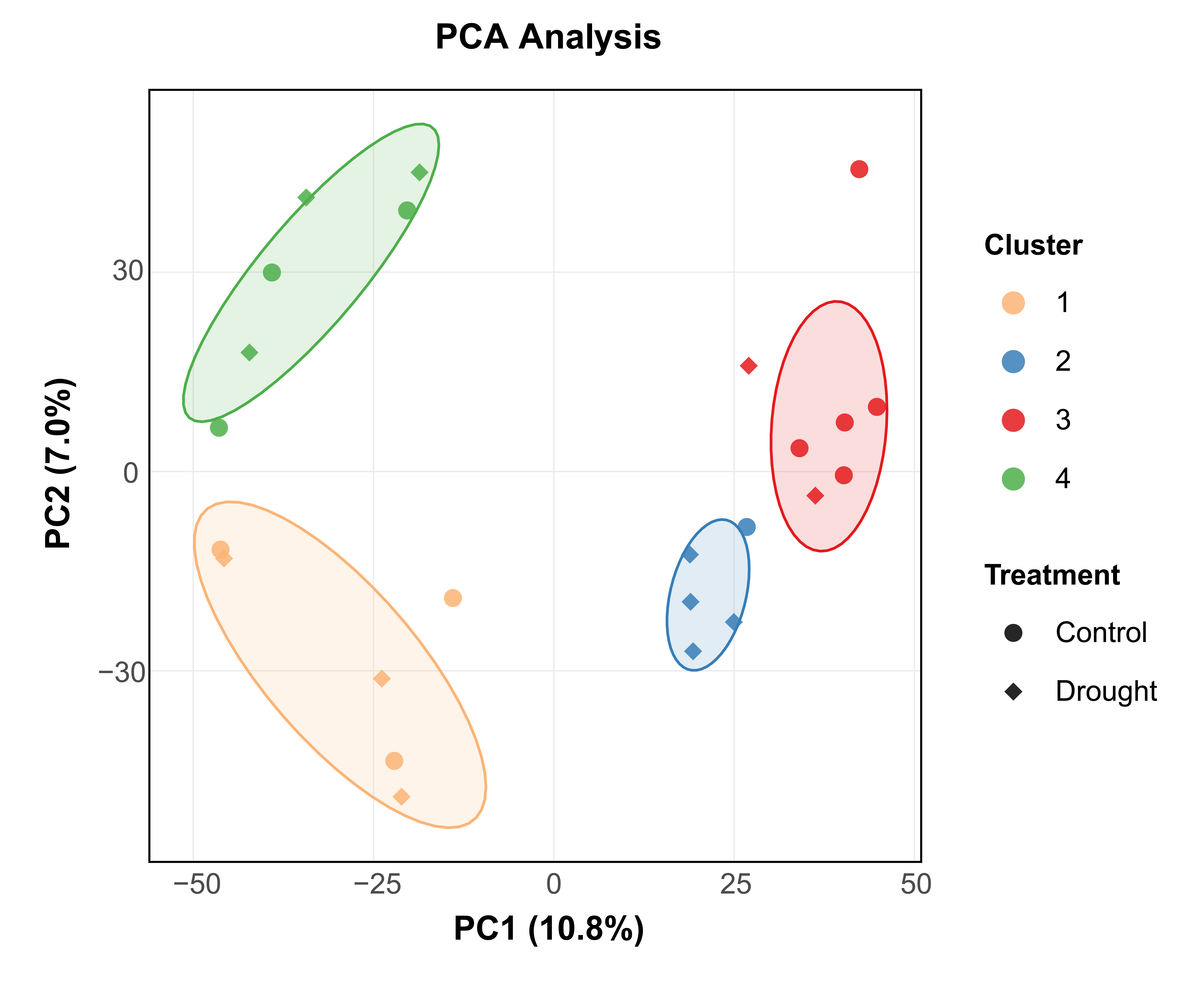

Supplement: Supplementary file 7 — Additional file 7: Figure S7. Clustering analysis of genome-wide DNA methylation profiles based on CG, CHG, and CHH methylation levels across all samples [file 12870_2026_8405_MOESM7_ESM.tif]

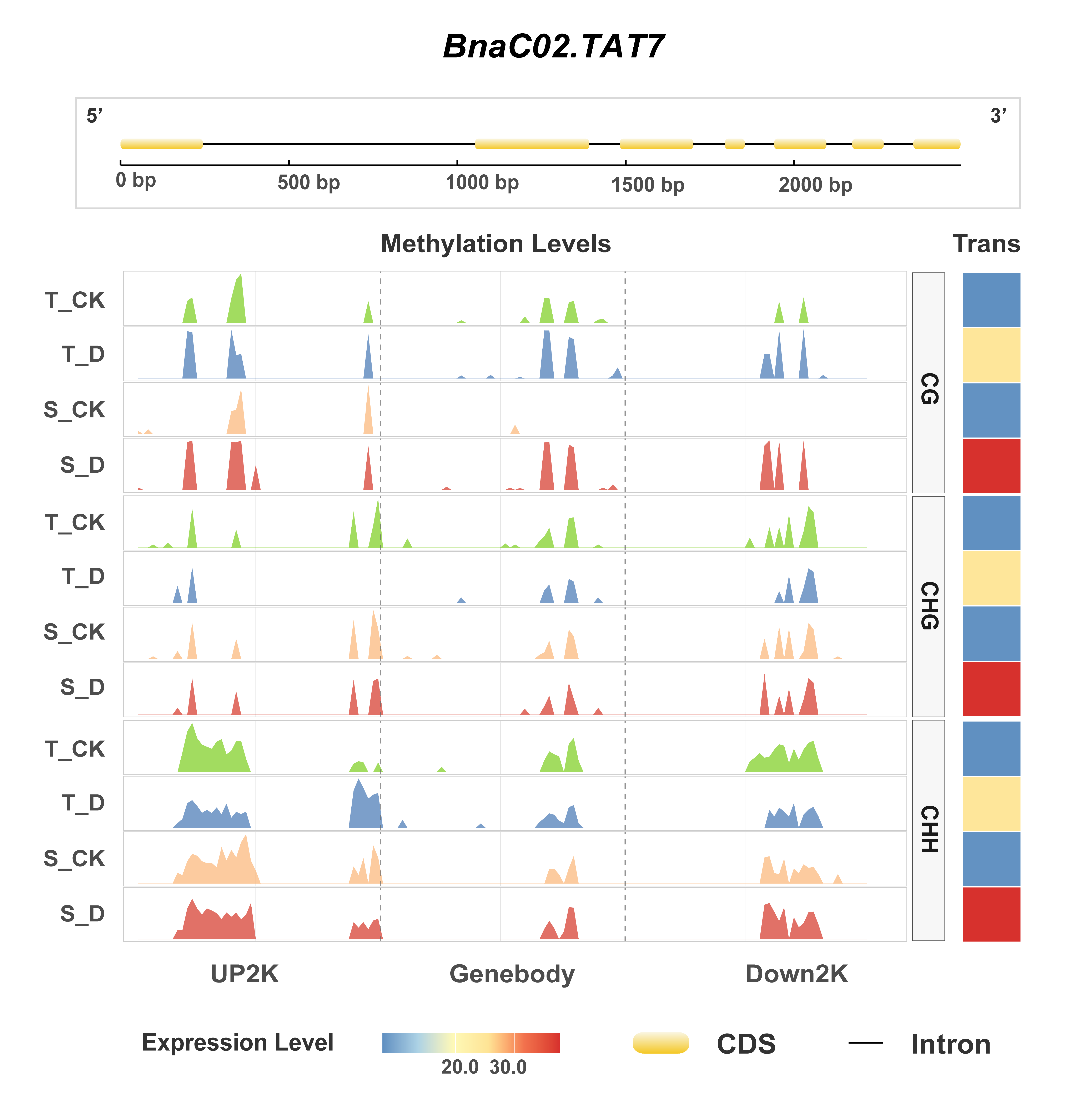

Supplement: Supplementary file 8 — Additional file 8: Figure S8. Multi-omics view of the BnTAT7 gene locus. The gene structure was drawn using GSDS (Gene Structure Display Server). DNA methylation patterns (CG, CHG, CHH) and expression levels are shown for sensitive and tolerant genotypes under control and drought conditions [file 12870_2026_8405_MOESM8_ESM.tif]

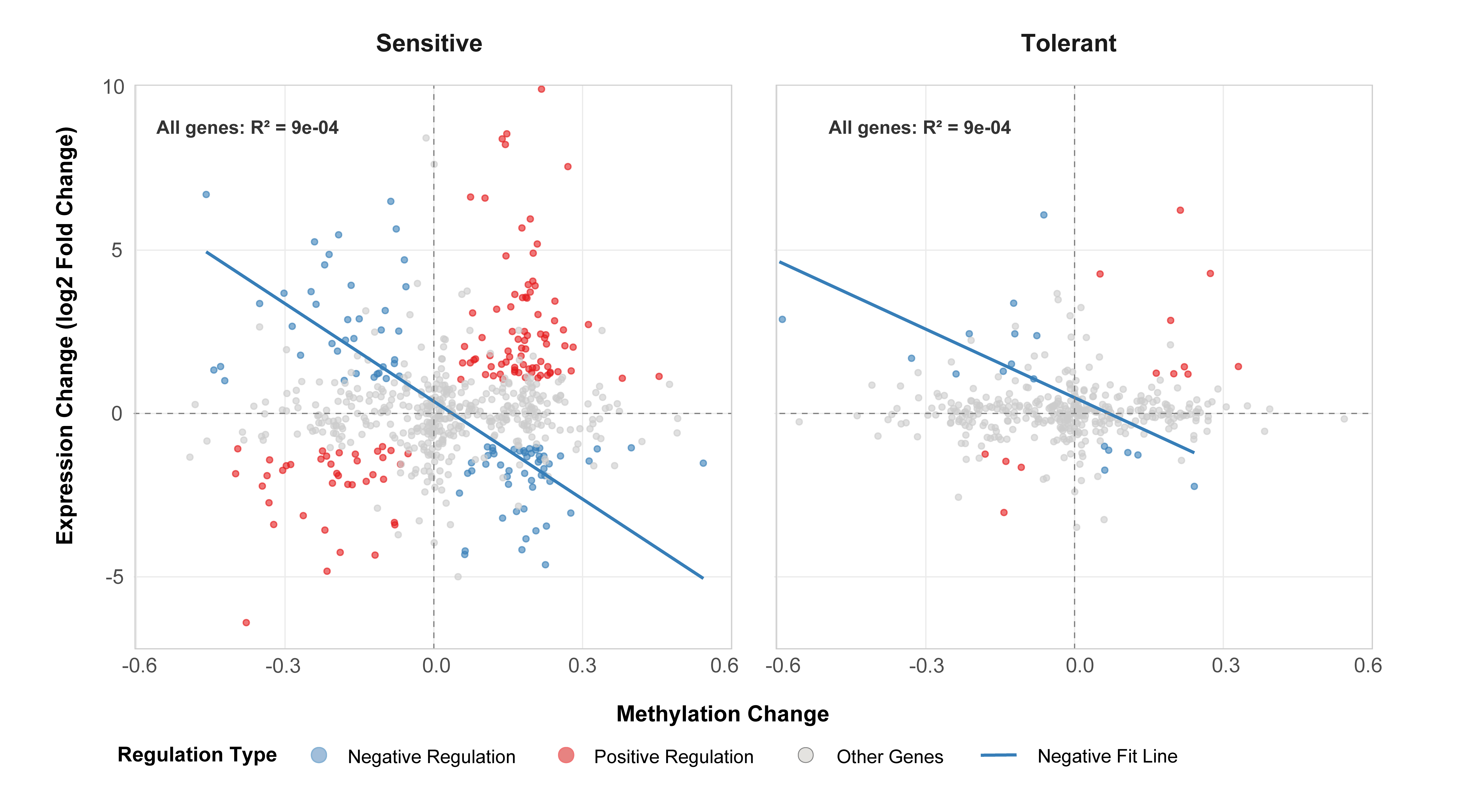

Supplement: Supplementary file 9 — Additional file 9: Figure S9. DNA methylation and gene expression relationships for differentially modified and expressed genes under drought stress. Correlation between DNA methylation changes (ΔMethylation > |0.05|) and gene expression changes (log2 fold change, padj < 0.05) in sensitive and tolerant genotypes. Analysis includes 3,206 genes that showed significant changes in both methylation and expression. Genes are categorized by their regulation pattern: negative regulation (blue, expression and methylation changes in opposite directions), positive regulation (red, expression and methylation changes in the same direction), and other genes (gray, no coordinated significant changes). Solid regression lines indicate the linear relationship specifically for negative regulation genes in each genotype. The top-left annotation displays the coefficient of determination (R²) for all genes combined, highlighting the weak genome-wide association [file 12870_2026_8405_MOESM9_ESM.tif]
